# Putative Iron Acquisition Systems in *Stenotrophomonas maltophilia*

**DOI:** 10.3390/molecules23082048

**Published:** 2018-08-16

**Authors:** V. Kalidasan, Adleen Azman, Narcisse Joseph, Suresh Kumar, Rukman Awang Hamat, Vasantha Kumari Neela

**Affiliations:** Department of Medical Microbiology and Parasitology, Faculty of Medicine and Health Sciences, Universiti Putra Malaysia, Serdang 43400 UPM, Selangor Darul Ehsan, Malaysia; petejuz@gmail.com (V.K.); adleen_azman@hotmail.com (A.A.); narcissemsjoseph@gmail.com (N.J.); sureshkudsc@gmail.com (S.K.); rukman@upm.edu.my (R.A.H.)

**Keywords:** *Stenotrophomonas maltophilia*, iron acquisition systems, iron-depleted, RAST server, NanoString Technologies, siderophores

## Abstract

Iron has been shown to regulate biofilm formation, oxidative stress response and several pathogenic mechanisms in *Stenotrophomonas maltophilia*. Thus, the present study is aimed at identifying various iron acquisition systems and iron sources utilized during iron starvation in *S. maltophilia*. The annotations of the complete genome of strains K279a, R551-3, D457 and JV3 through Rapid Annotations using Subsystems Technology (RAST) revealed two putative subsystems to be involved in iron acquisition: the iron siderophore sensor and receptor system and the heme, hemin uptake and utilization systems/hemin transport system. Screening for these acquisition systems in *S. maltophilia* showed the presence of all tested functional genes in clinical isolates, but only a few in environmental isolates. NanoString nCounter Elements technology, applied to determine the expression pattern of the genes under iron-depleted condition, showed significant expression for FeSR (6.15-fold), HmuT (12.21-fold), Hup (5.46-fold), ETFb (2.28-fold), TonB (2.03-fold) and Fur (3.30-fold). The isolates, when further screened for the production and chemical nature of siderophores using CAS agar diffusion (CASAD) and Arnows’s colorimetric assay, revealed *S. maltophilia* to produce catechol-type siderophore. Siderophore production was also tested through liquid CAS assay and was found to be greater in the clinical isolate (30.8%) compared to environmental isolates (4%). Both clinical and environmental isolates utilized hemoglobin, hemin, transferrin and lactoferrin as iron sources. All data put together indicates that *S. maltophilia* utilizes siderophore-mediated and heme-mediated systems for iron acquisition during iron starvation. These data need to be further confirmed through several knockout studies.

## 1. Introduction

Iron is a vital nutritional component for all living organisms, including pathogens, and is crucial for the preservation of cellular morphology, DNA and RNA biosynthesis, cellular growth and proliferation, catalysis of tricarboxylic acid cycle (TCA), electron transport chain (ETC), oxidative phosphorylation, nitrogen fixation and many more [1]. In order to successfully sustain an infective state in the human host, bacterial cells require a continuous supply of iron [2]. However, the mammalian host captures the freely available iron through high-affinity proteins such as transferrin (Tf), lactoferrin (Lf), ferritin (Fn) and heme (Hm) or hemoproteins and thereby protects itself from cellular damage by reactive oxygen species (ROS). As a result, the amount of iron is considerably reduced and the pathogens encounter a period of iron starvation upon invading their hosts [3]. In these circumstances, most pathogens sense the nutritional immunity imposed by the host, thereby expressing proteins associated with iron uptake for continual survival. In Gram negative bacteria, iron uptake occurs via (1) a siderophore-mediated system, (2) a hemophore-mediated system, (3) Tf/Lf receptors, and (4) ferrous-iron transport (Feo) [4].

Siderophore is a low molecular weight iron-complexing molecule characterized by high affinity and specificity for ferric iron (Fe^3+^) [5]. It is secreted by most bacteria including *Escherichia coli* [6] and fungi such as *Ustilago sphaerogena* [7] and *Aspergillus fumigatus* [8] under iron limited conditions to acquire iron from the host. Siderophores are considered to be pathogenic determinants, as these compounds chelate iron for proliferation in the host [9]. On the other hand, hemophores capture free heme or bind with heme from hemoglobin (Hb), or hemoglobin-haptoglobin (Hb-Hpt) complex or heme-hemopexin (Hm-Hpx) complex, and mediate further uptake into periplasm [4]. Tf/Lf receptors such as transferrin-binding proteins (TbpA and TbpB) and lacoferrin-binding proteins (LbpA and LbpB) are largely found in pathogenic *Neisseria* with the ability to directly interact with mammalian transferrin and lactoferrin for iron [10].

*S. maltophilia* is an emerging nosocomial, multiple-drug-resistant (MDR) and opportunistic pathogen, primarily from an environmental origin [11,12]. Infections are commonly seen among immunocompromised hosts, such as patients with invasive devices, prolonged hospitalization, and who are on broad spectrum antibiotics. Several infectious complications are associated with *S. maltophilia* ranging from bacteremia, bone and joint infections, wound infections, catheter related infections, meningitis, respiratory tract infections, endocarditis, typhlitis, biliary sepsis and peritonitis. *S. maltophilia* is also commonly isolated from the airways of cystic fibrosis patients [13], with an increased incidence seen in patients with hematological malignancy and among recipients of hematopoietic stem cell transplantation [14]. Furthermore, *S. maltophilia*’s complete genome revealed that the bacterium possesses a huge number of virulence factors and an antibiotics-resistance profile [15,16]. 

In *S. maltophilia*, iron has been shown to play an important role for biofilm formation, oxidative stress response, outer membrane proteins (OMPs) expression and other virulence factors via the regulation of ferric uptake regulation protein (FUR) [17]. Under iron-limited conditions, improved biofilm organization and formation, increased production of extracellular polymeric substances (EPS) and enhanced superoxide dismutase (SOD) activities were observed. Despite its clinical relevance and the role of iron in various pathogenic events, very little is known about the iron acquisition systems in *S. maltophilia* [18]. It was found that *S. maltophilia* uptakes ferrous iron through the Feo [19], and synthesizes siderophores under iron starvation to scavenge free ferric iron [20,21]. Nevertheless, genetic factors that possibly contribute to the siderophore-mediated iron uptake system in *S. maltophilia* are not well established [15]. When compared to most Gram-negative pathogens, the potential of heme, hemoproteins and other iron high-affinity ligands as nutrients during iron starvation has not been extensively studied in *S. maltophilia*. Therefore, this study is aimed at investigating the putative iron acquisition systems in *S. maltophilia* through various genotypic and phenotypic approaches with special focus on siderophore- and heme-mediated systems.

## 2. Results

### 2.1. Putative Iron Acquisition Systems in S. maltophilia

Targeted in-silico analysis of the four complete genomes of *S. maltophilia* strains (see Table 1), revealed the presence of shared iron acquisition and metabolism subsystems, with an additional system in some *S. maltophilia* strains. The subsystem information, generated through the Rapid Annotations using Subsystems Technology (RAST) server, is included in Supplementary Table S1. These subsystems include targets encoding iron siderophore sensor and receptor systems, heme, hemin uptake and utilization systems and the hemin transport system. In addition, encapsulating protein DyP-type peroxidase and ferritin-like protein oligomers were only detected in K279a. 

Ferric uptake regulation protein (FUR), which functions as a pleiotropic transcriptional regulator involved in the control of diverse cellular processes, such as iron homeostasis, oxidative stress responses, and the production of virulence factors, was observed across all the strains analysed. The targets and functional roles obtained from RAST server and locus tag (respective to *S. maltophilia* K279a) are listed in Table 2.

### 2.2. Distribution of Iron Acquisition Genes and Systems in S. maltophilia

In order to determine the distribution of iron acquisition genes and systems identified by the in-silico analysis in *S. maltophilia*, a total of 109 isolates (refer Table 1) obtained from different clinical and environmental sources were screened by PCR. All clinical isolates accounted for the 100% amplification for Hyp1, Hup, ETFb, TonB, DyP and FUR targets, while the remaining were as follows: FeSR (99%), HemO/HO (98.1%), FeSreg (96.2%), Rp2 (94.2%), HmuT (87.5%), HmuU (81.7%), FCR (53.8%), Htp (35.6%), ExbB (33.7%), HmuV (26.9%) and FeSS (25%). On the other hand, in environmental isolates, of the 17 targets tested, only eight showed amplification, which include Hyp1 (100%), Hup (100%), ETFb (100%), TonB (100%), DyP (100%), Fur (100%), FeSR (80%) and Rp2 (60%). The results from BLAST identities are shown in Table 2 and the information on the sequence homologue to the available genomes in the database are included in Supplementary Table S2.

### 2.3. Expression Profile of the Iron Acquisition System in S. maltophilia

**Figure 1:**
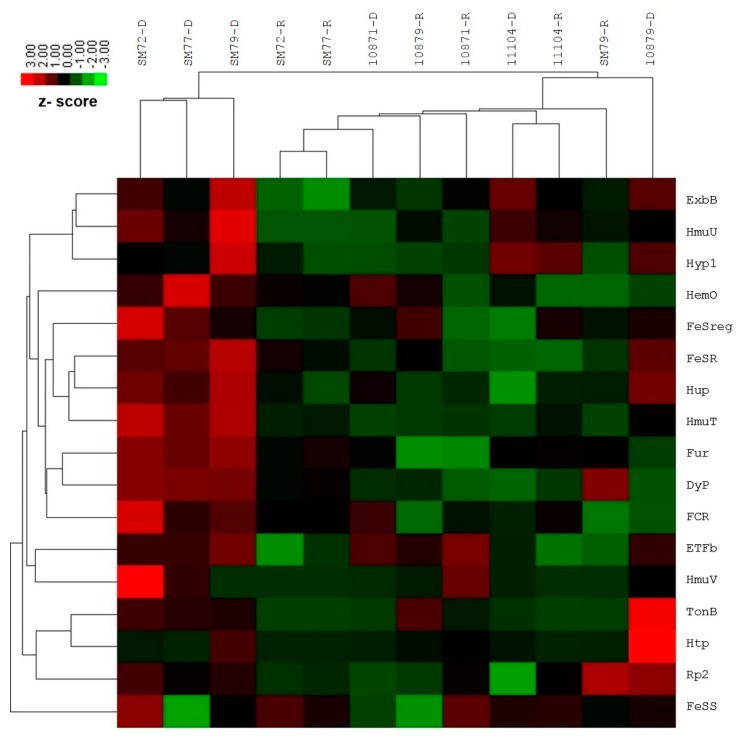
Heat map of nCounter (NanoString Technologies, Inc.) results comparing iron acquisition gene expressions of clinical (SM72, SM77, SM79) and environmental isolates (LMG10871, LMG10979, LMG11104) under iron-depleted and iron-repleted conditions. For clinical isolates, the up-regulated genes such as FeSR, Hup, HmuT, Fur, ETFb and TonB ranged from 1.00 to 3.00 under iron-depleted condition. While environmental isolates under both conditions, the targets remained neutral (no changes) or down-regulated but statistically insignificant. Red represents up-regulated targets under iron-depletion and green represents down-regulated targets.

The differential gene expression investigation by NanoString nCounter Elements showed significant upregulation for the following targets among the clinical isolates tested: FeSR (6.15-fold, *p* = 0.023), HmuT (12.21-fold, *p* = 0.005), Hup (5.46-fold, *p* = 0.014), ETFb (2.28-fold, *p* = 0.010), TonB (2.03-fold, *p* < 0.01) and Fur (3.30-fold, *p* = 0.003). The remaining functional targets exhibited no or slight fold changes, which is statistically insignificant: FeSreg (2.40-fold), HemO/HO (3.34-fold), HmuU (8.14-fold), HmuV (2.34-fold), Hyp1 (3.16-fold), Rp2 (1.14-fold), ExbB (3.64-fold), Htp (1.28-fold), FCR (3.73-fold) and DyP (2.35-fold). One siderophore-mediated target, FeSS (−1.36-fold) was found to be down-regulated; however, this was not statistically significant. In the case of environmental isolates, none of the functional targets showed statistically significant changes in the gene expression for both iron conditions tested. The average normalized grouped data and the fold changes of gene expression under iron-depleted and iron-repleted conditions for each target, together with *p*-values, are listed in Supplementary Table S3. The differentially expressed targets during iron-depleted and iron-repleted conditions were generated using nSolver software. The agglomerative cluster of the heat map with a dendrogram tree showed an obvious clustering of up-regulated genes for clinical isolates (SM72, SM77, and SM79) that ranged from 1.00 to 3.00 under the iron-depleted condition, as illustrated in Figure 1. Red indicates an increase in gene expression, and green indicates a decrease in gene expression. This figure, which represents data from three clinical isolates and three environmental isolates tested against 17 targets, showed at least a two-fold differential expression based on normalized grouped counts under the different iron conditions tested.

### 2.4. Siderophore Production and Its Chemical Nature

**Figure 2:**
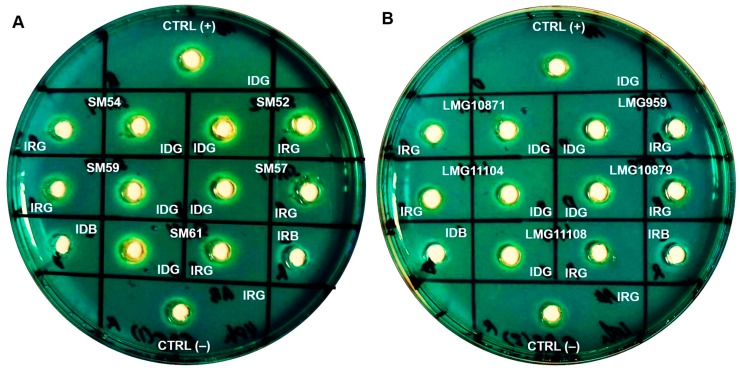
Representation of CAS agar diffusion (CASAD) agar plates showing zone of orange halo after 72 h incubation at room temperature. (**A**) Clinical isolates: SM52, SM54, SM57, SM59 and SM61; (**B**) Environmental isolates: LMG959, LMG10871, LMG10879, LMG11104 and LMG11108. Key: CTRL (+): Positive control; CTRL (−): negative control; IDG: iron-depleted growth; IRG: iron-repleted growth; IDB: iron-depleted uninoculated broth; IRB: iron-repleted uninoculated broth.

Cultures that were grown to stationary phase for 48 h were subjected to an optical density (OD) measurement at 600 nm. The OD readings (mean, SD) of *S. maltophilia* grown in iron-depleted BHI broth were lower (1.007, 0.276), than those obtained in iron-repleted BHI broth (1.329, 0.485), showing sufficient iron starvation. Siderophore activity was observed in the cell-free culture supernatants of ten clinical and five environmental isolates tested. All isolates exhibited a prominent zone of an orange halo surrounding the well which was inoculated with the supernatants of cultures grown under iron-depletion, compared to the slight/lesser zone under iron-repleted conditions. The zone size and intensity of the orange halo was lower for environmental isolates when compared to clinical isolates, as seen in Figure 2. Arnow’s assay, performed to identify the chemical nature of the siderophores, revealed that *S. maltophilia* secreted catechol-type as it formed a yellow color in nitrous acid, which then turned to pink-red when excess sodium hydroxide was added (data not shown). On another note, through liquid CAS assay, CS17 was found to produce greater percentage of siderophores (30.8%) when grown under iron-depleted compared to iron-repleted conditions (<5%) (*p* < 0.05). However, LMG10879 showed only 4% siderophore production, but this was not statistically significant. 

### 2.5. Iron Source Utilization during Iron Starvation

**Figure 3:**
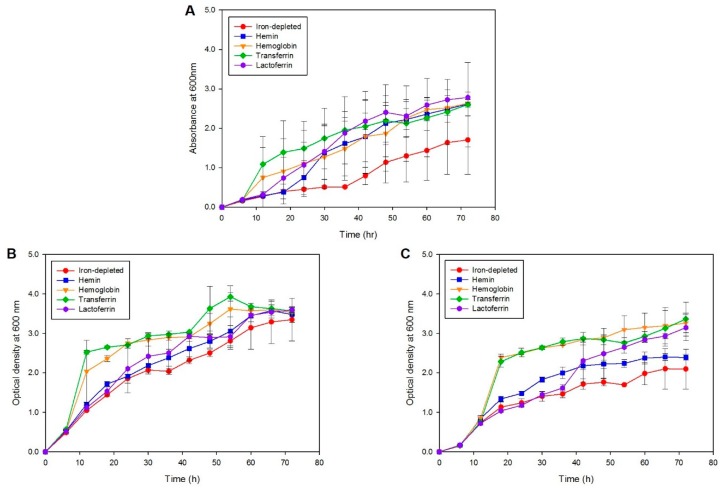
Growth kinetics curve (**A**) Positive control: *N. meningitidis*, (**B**) Clinical isolate: SM77 and (**C**) Environmental isolate: LMG10879. The symbol shape represents the mean reading and the error bars represent the standard deviation. Iron-depleted versus respective iron-repleted sources, post-hoc test (Duncan’s Method) showed *p* < 0.05. The growth in the iron-depleted condition remains low in comparison to the iron-replete condition.

To identify the iron sources utilized by *S. maltophilia* during iron starvation, one clinical (SM77) isolate and one environmental (LMG10879) isolate grown in the presence of different iron sources were investigated. As seen in Figure 3, although growth was observed for all iron sources, the maximum and fast replication was seen in the presence of transferrin (*p* < 0.001), followed by hemoglobin (*p* < 0.001) for both clinical and environmental isolates. Both strains utilized similar iron sources for growth during iron starvation; however, the clinical isolate exhibited a higher growth rate compared to environmental isolates. The mean OD readings for the positive control, *N. meningitidis*, SM77, and LMG10879 under the iron-depleted and iron-repleted conditions, are shown in Supplementary Table S4. On the other hand, the growth under iron-depletion remained low, underscoring the importance of iron for the growth and replication of bacterial cells. From 54 h onwards, the growth under iron-depletion slowed and was similar to iron-repletion, indicating that the utilization of supplemented iron and the reach of the stationary phase. For the isolates from both sources, a decline in growth was observed from 72 h onwards.

## 3. Discussion

The in-silico approach using the RAST server revealed two prominent subsystems for iron acquisition in *S. maltophilia* strains K279a, R551-3, D457 and JV3, which suggests that the bacteria may acquire the iron source through siderophore- and/or heme-mediated iron acquisition systems. These systems are regulated by FUR, which is expressed during oxidative stress response, such as for iron starvation, as described in an earlier study [17]. Among the 17 putative functional targets tested which are involved in iron acquisition, only eight showed a positive signal in PCR for environmental strains. The environmental isolates contained fewer of the sequences predicted to be involved in iron transport and homeostasis compared to clinical isolates as observed by the molecular study. The genome of clinical *S. maltophilia* strain K279a [16] and environmental *S. maltophilia* strain R551-3 [30] showed a large variation in the sequences, which could suggest the differences in the mechanisms for pathogenicity in the human host [12]. On the contrary, a recent study showed that the coding DNA sequence (CDS) composition and the distribution of virulence genes among clinical and environmental *S. maltophilia* is not distinguishable [31]. Therefore, it is hypothesized that the presence and absence of the functional targets among clinical and environmental isolates could also be influenced by the molecular diversity of *S. maltophilia* strains and the nature of the availability of the source of iron in a specific environment [32]. 

In this study, iron removal was achieved by using 2,2′-dipyridyl as metal chelating ligand [33]. It forms charged complexes with metal cations, and this property is useful in the synthesis of iron-dipyridyl. The bacterial cells treated with 2,2′-dipyridyl become pink in color because of the uptake of the chelator and formation of Fe(II) complex inside the cell [34]. 2,2′-dipyridyl can cause iron depletion and the depression of iron-regulated proteins and siderophores, even in the presence of normally repressive levels of iron in the medium. However, it is important to note that, 2,2′-dipyridyl can cause the loss of cell viability, thus chelators to cause iron limitations must be monitored carefully. The suggested final concentration of 2,2′-dipyridyl to be added into a media is 100 to 400 µM [35]. During optimization, the chelator did not render iron removal in a concentration of 50 µM, while at 200 µM bacterial growth was inhibited. Furthermore, *S. maltophilia* was grown for 48 h, as different levels of regulation were noted at the onset of stationary phase in Gram-negative bacteria such as sigma factors [36] and expression of iron-regulated outer membrane protein (IROMP) [17].

Among the 17 functional targets analysed for expression using the NanoString nCounter Elements, seven targets showed significant fold changes in clinical isolates, indicating the derepression of these targets; although other targets showed some degree of fold changes, no significant up or down regulation was observed. The environmental isolates did not show any significant differential expression for the targets tested, when grown under both iron-depleted and iron-repleted conditions. As observed in molecular screening, the targets that participated in iron acquisition and metabolism are not well manifested among environmental isolates. Moreover, the existence of iron uptake mechanisms is certainly advantageous to the growth of pathogenic bacteria under limited iron availability [32]. Specifically, a considerable difference might be seen in the type of iron transporters and iron sources utilization among different bacteria. It is worthwhile to note that the expression of various iron acquisition system studied herein, under laboratory conditions, is not well-established among environmental isolates. Further validation of the assay using real-time quantitative PCR (RT-qPCR) was not necessary, as NanoString nCounter Elements results were found to be as accurate as RT-qPCR in bacterial gene expression study [37].

The discrepancy in the gene expression profile among clinical and environmental isolates may also be attributed to the biological origin of the strains. As the environment contains a high amount of nutrients such as iron, particularly in the soil [38], the necessity of expressing special systems to acquire iron may not be vital. Thus, a low concentration of siderophores is usually detected in soil extracts [39]. Under an extreme iron demand in pure culture, pseudomonads distribute most of their carbon and ATP to synthesize siderophore. The degree of iron stress experienced by the environmental bacteria in the rhizosphere is much lower than what occurs in pure cell cultures. Moreover, the necessity for siderophore production in the rhizosphere depends largely on the effectiveness of plant iron stress responses, which is important to raise the iron availability to both plants and rhizosphere bacteria. 

In the present study, siderophore detection using CASAD assay showed a prominent zone of a halo under iron-depletion in comparison with the iron-replete condition. This suggests that extracellular siderophore is secreted during iron starvation to scavenge the free iron available in the blue-green agar. However, a notable variation of intensity among the clinical and environmental isolates was observed. In support of this, the percentage of siderophore production was investigated through liquid CAS, and the clinical isolate produced a greater amount of siderophore compared to environmental isolate when grown under iron-depleted condition. Our results are in agreement with earlier studies, which reported that the environmental strain did not produce siderophores or produced very minimal amounts compared to clinical isolates [20,40]. Arnow’s assay concluded that the *S. maltophilia* isolates tested in this study are catechol-type siderophore producers, as reported previously [21,41,42]. *S. maltophilia* was reported to produce hydroxamate-type ornibactin siderophore [43]. The data from the molecular and phenotypic studies support the notion that *S. maltophilia* uses siderophore-mediated iron acquisition system for obtaining iron.

In order to identify the iron source utilized by *S. maltophilia,* an iron assimilation assay was performed. *N. meningitidis* was chosen as a positive control because it is a well-established model for iron uptake from heme, lactoferrin, and transferrin [44]. The growth in iron-depleted media remained low throughout the kinetic study in comparison to the iron-repleted conditions. Hemoglobin and transferrin stimulated the growth of both strains tested under iron-depleted conditions, with hemin and lactoferrin having less effect in enhancing the growth of SM77 and LMG10879. SM77 showed sufficient growth under iron-replete conditions compared to iron-depleted ones, whereas LMG10879 showed clear differences in iron utilization patterns. This suggests that the clinical isolate utilizes of even minor traces of iron which could be present in the medium. The comparatively lower growth of LMG10879 than SM77 in the presence of different iron sources explains the low number of amplified targets in PCR and non-expression of iron uptake genes in the environmental isolates during iron depletion. Overall, *S. maltophilia* utilizes iron sources such as ferric iron or other iron-containing proteins such as hemoglobin, lactoferrin, and transferrin for cellular growth and proliferation.

The utilization of hemin and hemoproteins by *S. maltophilia* may be contributed by the heme uptake locus (*hmu*) detected in-silico and also through PCR in this study, as these similar genes *hmuRSTUV* were found in *Yersinia pestis* [45,46,47]. The hemoprotein-receptor-based system encoded by *hmuRSTUV* operon is used for the utilization of both hemin and other hemoproteins in *Y. pestis.* All of the other three targets were observed in *S. maltophilia*, except for HmuR and HmuS. This indicates that both *S. maltophilia* and *Y. pestis* may use a similar mechanism in acquiring iron from hemoproteins. Hemin uptake system found in *Yersinia enterocolitica* is found to pose similarities with other TonB-dependent systems in Gram-negative bacteria [48]. Thereby, the similarity between the heme-mediated systems of *S. maltophilia* with *Yersinia* spp. in this study is affirmed.

The expression of siderophore- and heme-mediated system under the iron-depleted condition in term of genotypic and phenotypic profiles reveals that it is possible to elucidate how *S. maltophilia* could establish its pathogenicity upon invasion. FeSR acts as a receptor protein, which allows the internalization of an iron-bounded siderophore complex, which must pass the outer membrane (OM) and cytoplasmic membrane (CM) before reaching the cytoplasm [49]. The siderophore detected in CASAD assay could potentially scavenge not only the ferric iron, but is also capable of delivering iron-saturated Tf, Lf, or hemin and hemoproteins, investigated through iron assimilation assay [50]. For a heme-mediated system, the intake of hemin and hemoproteins from the extracellular space into the cytoplasm occurs via HmuTUV systems [51]. The hemin and hemoglobin utilization revealed how *S. maltophilia* could potentially utilize other iron sources apart from ferric iron. This would give an indication, upon bloodstream infection with *S. maltophilia*, that bacterial multiplication within the blood stream is possible, as a unit of packed erythrocyte contains approximately 200 mg of iron, which serves an alternative source of iron [52]. In support of this, blood transfusion for an anemic patient admitted to the medical-surgical-trauma intensive care unit (ICU) was found to be associated with nosocomial infections such as pneumonia, bacteremia, sepsis, and cystitis [53].

## 4. Materials and Methods

### 4.1. Bacterial Strains, Identification and Culture Conditions

A total of 103 clinical isolates (referred to as SM in Table 1) were isolated from blood, swab, urine, tracheal aspirates, cerebrospinal fluid (CSF), pus swab, nasopharyngeal aspirates (NPA) and sputum including CS17 (clinical invasive) and CS24 (clinical non-invasive) as reference strains obtained from the laboratory culture collections (Department of Medical Microbiology and Parasitology, Universiti Putra Malaysia, Serdang, Selangor, Malaysia) were used in this study. *S. maltophilia* ATCC13637 (clinical) purchased from the American Type Culture Collection (ATCC, Manassas, VA, USA) and five environmental isolates, LMG959 (rice paddy), LMG10871 (soil), LMG10879 (rice paddy), LMG11104 (*Cichorium intybus*, rhizosphere tuberous roots) and LMG11108 (Triticum, roots); purchased from Belgian Coordinated Collections of Microorganisms (BCCM) (Laboratorium voor Microbiologie, Universiteit Gent, Belgium) were also studied. The isolates were incubated aerobically for 24 h at 37 °C for clinical and 30 °C for environmental isolates.

All isolates were previously identified as *S. maltophilia* using standard biochemical assays, API 20 NE (bioMerieux, Marcy-l’Étoile, France) and confirmed by the VITEK^®^ Mass Spectrometry System [25,26]. Besides this, the isolates were morphologically identified by culture characteristics on Columbia agar with 5% sheep blood (Isolac, Selangor, Malaysia) and Gram morphology. The isolates were re-confirmed genotypically by species-specific polymerase chain reaction (SS-PCR) as previously described [54].

### 4.2. In-Silico Analysis of Putative Iron Acquisition Systems

The complete genome sequences of all four *S. maltophilia* strains, K279a, R551-3, D457 and JV3 (refer Table 1) were downloaded from the National Centre for Biotechnology Information (NCBI, Bethesda, MD, USA) Genbank (www.ncbi.nlm.nih.gov/genome/browse/). The genomes were annotated by Rapid Annotations using Subsystem Technology (RAST) server (http://rast.nmpdr.org/) [55]. RAST is a fully automated annotation service that produces gene functions and an initial metabolic reconstruction. Iron acquisition genes and gene clusters were identified by intrinsic RAST subsystem profiling for each genome as well as through, gene homologs search by Basic Local Alignment Search Tool (BLAST). Comparative genomics in SEED viewer (Genome Viewer) were used to confirm the identification and conservation of putative iron acquisition genes within *S. maltophilia* genome sequences [56].

### 4.3. Screening of Iron Acquisition Systems by Polymerase Chain Reaction (PCR)

PCR primers targeting the different putative iron acquisition genes and gene clusters were designed. All primers were derived from consensus sequences of four complete genome of *S. maltophilia* strains aligned through multiple sequence alignment (CLUSTALW) program using Molecular Evolutionary Genetics Analysis (MEGA 6) software [57]. Primers were designed using PrimerQuest Tool in order to select the optimal primers for PCR assay and further comprehensive oligonucleotide analysis was conducted using OligoAnalyzer 3.1 [58]. The sequences of the primers, targeted functional roles and the amplification parameters used for each set of primers are listed in Table 3.

The genomic DNA was extracted from both clinical and environmental isolates of *S. maltophilia* using Wizard^®^ Genomic DNA Purification Kit as per the manufacturer’s protocol (Promega, Madison, WI, USA). All PCR mixtures were prepared using 25 µL per tube containing 12.5 μL of EconoTaq^®^ PLUS GREEN Master Mix, 2X (Lucigen Corporation, Middleton, WI, USA); 10 μM of forward and reverse primers; 10 ng of DNA template and 10.5 μL nuclease-free water. Target amplification was carried out in a thermal cycler (MyCycler Personal Thermal Cycler, BioRad, Hercules, CA, USA) programmed for one step of initial denaturation at 95 °C for 2 min followed by 30 cycles comprised of denaturation at 95 °C for 30 s, primer annealing at 50–51 °C for 30 s (refer Table 3), primer extension at 72 °C for 1 min, and a final extension at 72 °C for 5 min. The purified PCR products with corresponding primer pairs were sequenced through commercial company (1st Base Sdn. Bhd., Selangor, Malaysia) and analyzed using Biology Workbench 3.2 (SDSC Biology Workbench: http://workbench.sdsc.edu/). The sequences were then subjected to Standard Nucleotide BLAST (https://blast.ncbi.nlm.nih.gov/Blast.cgi) to determine the percentage of query cover and identities as well as features against *S. maltophilia*’s complete genome.

### 4.4. Bacterial Culture under Iron-Depleted and Iron-Repleted Conditions

A few single colonies were picked from the agar plates and inoculated into brain–heart infusion (BHI) broth. Followed by overnight incubation, cultures were centrifuged for 5 min at 10,000 rpm. The cell pellets were resuspended in phosphate buffered saline (PBS), centrifuged and washed twice. The cell suspensions in PBS were adjusted to 0.2 with an Eppendorf BioPhotometer Plus (Hamburg, Germany) at an optical density (OD) of 600 nm. One milliliter of a standardized suspension was used to inoculate the media prepared under two conditions. An iron-depleted condition was achieved by adding an iron chelator, 100 µM 2,2′-dipyridyl (DIP) (Sigma Aldrich, Darmstadt, Germany) to BHI broth (BHI-DIP), while the iron-repleted condition was further defined by the addition of 100 µM ferric chloride (Sigma Aldrich) to the BHI-DIP. The tubes were incubated aerobically (37 °C for clinical and 30 °C for environmental isolates) for 48 h on an incubator shaker (Model IKA^®^ KS 4000 i control, IKA^®^ Works (Asia) Sdn Bhd, Selangor, Malaysia) at 200 rpm to ensure stationary phase bacterial growth [21]. All glassware were treated with 3M HCl followed by extensive washing with deionized water to remove any iron from the labware before proceeding with the experiments [59].

### 4.5. Gene Expression of Iron Acquisition Systems by NanoString Technologies

It was hypothesized that the expression of *S. maltophilia* iron acquisition genes will be enhanced under iron-depleted conditions to encounter iron starvation. Based on in-silico analysis and positive molecular screening, three clinical isolates (SM72, SM77 and SM79) and three environmental isolates (LMG10871, LMG10879 and LMG11104) that amplified most functional targets were selected for gene expression study. Total RNA was extracted from each culture conditions using Agilent Total RNA Isolation Mini Kit-Bacteria (Agilent Technologies, Santa Clara, CA, USA) as per the manufacturer’s protocol. The extracted total RNA was used for expression study using nCounter^®^ Elements technology (NanoString Technologies, Inc., Seattle, WA, USA). The technology is based on molecular barcoding and digital quantification of target RNA sequences through the use of nCounter Elements TagSet and target-specific oligonucleotide probe pairs (Probe A and B). The nCounter Elements probes consist of the targets’ name, GenBank accession numbers, a position of the targets, target sequences and melting temperature (T_m_) for both Probe A and Probe B as shown in Supplementary Table S4.

The nCounter assay comprising of three steps including hybridization, sample processing, and digital data acquisition were performed as per the manufacturer’s instructions. The components including hybridization buffer, code set and RNA samples were added into a strip tube [60]. The hybridization was performed in a thermal cycler (Turbocycler2, Blue-Ray Biotech, Taipei City, Taiwan) programmed for 16 cycles at 67 °C for 60 min and holding at 4 °C for infinity. The samples were then processed by placing the strip tubes into the automated nCounter Prep Station with reagents and consumables from the nCounter Master Kit. After the purification and immobilization were completed, the cartridge was taken out from the Prep Station and placed into the Digital Analyzer for digital data counting. The cartridges were scanned at a maximum resolution of 555 Field of View (FOV) [61]. The fold changes in expression under iron-depleted and iron-repleted conditions were analyzed based on “all pairwise ratios” of the normalized grouped data using nSolver™ Analysis software version 3.0, considering iron-depleted as the baseline condition for comparison. Four parameters were used as quality control (QC) which include imaging QC, binding density QC, positive control linearity QC and positive control limit of detection QC [62]. Both positive control and housekeeping normalization were used to normalize all platform associated sources of variations.

### 4.6. Siderophore Detection Using CASAD and Colorimetric Assays

Extracellular siderophore production was examined using the CASAD method by modifying the classical CAS plate as described previously [63]. Based on in-silico analysis and positive molecular screening for most of the functional targets, ten randomly selected clinical isolates (CS17, CS24, ATCC13637, SM49, SM50, SM52, SM54, SM57, SM59, and SM61) and five environmental isolates (LMG959, LMG10871, LMG10879, LMG11104, and LMG11108) were used for the assay. Briefly, 5 mm diameter holes were punched on the CASAD agar plate and each hole was filled with 70 μL (35 μL twice) of cell-free-supernatant containing secreted extracellular siderophore [32]. The plates were incubated at room temperature for 72 h. Formation of orange halo zone around the holes inoculated with cultures indicates positive reaction. *Acinetobacter baumannii* ATCC 19606 was used as positive control, while uninoculated BHI broth (both iron-depleted and iron-repleted) served as the negative control.

The liquid CAS assay was performed as described previously [64], with *A. baumannii* ATCC 19606 positive control. To estimate the quantity of siderophores produced, 500 μL of culture supernatant was added to 500 μL CAS solutions. Upon 30 min of incubation at room temperature, the absorbance was read at 630 nm [64,65]. The BHI-DIP was used as a blank, BHI-DIP plus CAS assay solution plus shuttle as a reference (r) and culture supernatant as a sample for testing (s). The percentage of iron-binding compounds of the siderophore type was calculated by subtracting the sample absorbance (*A_s_*) values from the reference (*A_r_*). Siderophore units are defined as $\frac{A_{r}-A_{s}}{A_{r}}\;\times \;100$ = percent siderophore units [35,66]. Percentages of siderophores units less than 10 were considered as negative, which is indicated by no change in the blue color of CAS solution. 

The chemical nature of siderophores (phenolic-type and/or hydroxamate-type) produced in the cell-free supernatant was detected using the colorimetric assays described by Atkin [67] and Arnow [68] respectively. *Escherichia coli* ATCC 8739 (aerobactin hydroxamate-type siderophore producer) [69] was used as positive control for Atkin’s method, while *A. baumannii* ATCC 19606 (catecholate-type siderophore producer) [70] served as a positive control for Arnow’s method.

### 4.7. Iron Utilization Kinetics Using Liquid Assimilation Assay

To determine the iron source utilized by *S. maltophilia* during iron starvation, one clinical isolate (SM77) and one environmental isolate (LMG10879) were investigated, based on positive molecular screening and gene expression study for most of the functional targets. The BHI-DIP was supplemented with other iron sources to make the BHI broth iron-repleted. Iron sources used herein include 10 µM hemin chloride (bovine) (MP Biomedicals, Santa Ana, CA, USA) (dissolved in 1.4 M ammonia hydroxide), 2.5 µM hemoglobin (human) (Sigma Aldrich) (dissolved in deionized water), 1 µM iron-saturated lactoferrin (human) (Sigma Aldrich) (dissolved in PBS) and 5 µM iron-saturated transferrin (human) (MP Biomedicals) (dissolved in deionized water) [71,72]. The iron utilization kinetics was measured by observing the turbidity of the culture. 100 µL of culture was filled into the UV-Vis cuvette and turbidity was measured with an Eppendorf BioPhotometer Plus (Hamburg, Germany) at the optical density (OD) of 600 nm.

The bacterial growth was tested every 6 h up to 72 h for growth kinetics measurement. *Neisseria meningitidis* obtained from Department of Medical Microbiology and Immunology, Universiti Kebangsaan Malaysia Medical Centre (UKMMC) grown anaerobically on chocolate agar II (Isolac, Selangor, Malaysia) in a candle jar served as the positive control [73,74]. Bacterial growth in BHI iron-depleted broth was used as the negative control to show the comparison of growth kinetics between two conditions tested.

### 4.8. Data and Statistical Analysis

For gene expression study, the distribution of the *t*-statistic was calculated by the Welch-Satterthwaite equation using nSolver™ Analysis software version 3.0. The *p*-values were set at (*p* < 0.05) to be considered statistically significant, as the lower *p*-value, the stronger the evidence that the two different groups have different expression levels. On the other hand, the data obtained from iron assimilation assay were analyzed through SigmaPlot version 12.5 and statistically significant data was determined by using One Way Repeated Measures Analysis of Variance (One Way RM ANOVA) (*p* < 0.05). To determine the significance in the difference of means among the iron sources supplemented, all pairwise multiple comparison procedures (Duncan’s Method) were performed.

## 5. Conclusions

In conclusion, it is revealed that *S. maltophilia* expresses two putative iron acquisition systems—the iron siderophore sensor and receptor system and the heme, hemin uptake and utilization systems/hemin transport system—during iron starvation. It is indicated that siderophore-based iron uptake is mediated through FeSR and TonB, while the heme-mediated uptake may involve targets such as HmuT, Hup, and ETFb. Both clinical and environmental isolates produced catechol-type siderophores and utilized all iron sources tested, such as hemin, hemoglobin, transferrin, and lactoferrin. This study is the first step towards understanding iron acquisition systems in *S. maltophilia* focusing on siderophore- and heme-mediated systems.

A major limitation of the study was that the role of each gene in iron acquisition during starvation could not be established. However, in future studies, a mutant construction for each gene will be considered to understand the roles of differentially expressed genes during iron starvation. Further investigation on the heme acquisition system would be able to provide firmer evidence on how *S. maltophilia* utilizes heme or hemoproteins.

## Figures and Tables

**Table 1:** *S. maltophiia* strains for in-silico, clinical and environmental isolates used in this study.

Strain I.D.	Biological Source	Geographical Source	GenBank Accession Number	References
**In-silico**				
K279a	Clinical (Blood infection)	Bristol, UK	AE016879	[16]
R551-3	Environmental (Poplar tree endophyte)	Washington, USA	CP001111	[22]
D457	Clinical	Mostoles, Spain	HE798556	[23]
JV3	Environmental (Rhizosphere)	Brazil	CP002986	[24]
**Clinical**				
SM1 to SM 101	Various specimens	Malaysia	—	[25,26]
CS17	Blood	Malaysia	—	[26]
CS24	Wound swab	Malaysia	—	[26]
ATCC13637	Pleural fluid of a patient with oral carcinoma	Stafford, England	CP008838	[27]
**Environmental**				
LMG959	Rice paddy	Japan	—	[28]
LMG10871	Soil	Japan	—	[28]
LMG10879	Rice paddy	Japan	—	[28]
LMG11104	Roots	Unknown	—	[29]
LMG11108	Roots	Unknown	—	[29]

**Table 2:** Functional roles (RAST server), their abbreviations, locus tags respective to *S. maltophilia* K279a genome and BLAST identities respective to *S. maltophilia* K279a, D457 and 13637 genomes.

Targets *^a^*	Functional Role *^a^*	Locus Tag *^b^*	BLAST Identity *^c^*
**Subsystem: Iron siderophore sensor & receptor systems**			
FeSreg	Sigma factor ECF subfamily	SMLT_RS12950	98% (13637)
FeSR	Iron siderophore receptor protein	SMLT_RS18575	99% (D457)
FeSS	Iron siderophore sensor protein	SMLT_RS18580	99% (K279a)
**Subsystem: Heme, hemin uptake and utilization systems in Gram-positives**			
HemO/HO	Heme oxygenase, associated with heme uptake	SMLT_RS18565	100% (13637)
HmuV	Heme ABC transporter, ATPase component	SMLT_RS11325	99% (K279a)
Hyp1	Hypothetical protein related to heme utilization	SMLT_RS19415	98% (1337)
HmuU	Heme ABC transporter, permease protein	SMLT_RS11320	92% (K279a)
HmuT	Heme ABC transporter, cell surface heme and hemoprotein receptor	SMLT_RS11315	97% (D457)
**Subsystem: Heme, hemin uptake and utilization systems in Gram-negatives**			
Rp2	Outer membrane receptor proteins, mostly Fe transport	SMLT_RS18050	95% (D457)
Hup	Hemin uptake protein	SMLT_RS03780	100% (D457)
ETFb	Electron transfer flavoprotein, beta subunit	SMLT_RS03080	100% (D457)
TonB	Ferric siderophore transport system, periplasmic binding protein	SMLT_RS21345	99% (D457)
ExbB	Ferric siderophore transport system, biopolymer transport protein	SMLT_RS07890	99% (K279a)
Htp	Hemin transport protein	SMLT_RS03790	99% (K279a)
FCR	TonB-dependent hemin, ferrichrome receptor	SMLT_RS03785	99% (K279a)
**Subsystem: Encapsulating protein DyP-type peroxidase and ferritin-like protein oligomers**			
DyP	Predicted dye-decolorizing peroxidase, encapsulated subgroup	SMLT_RS00875	95% (K279a)
**Subsystem: Oxidative stress**			
Fur	Ferric uptake regulation protein (FUR)	SMLT_RS09600	96% (K279a)

*^a^* The abbreviations of the targets and name of the functional roles are derived from RAST server; *^b^* Corresponding locus tag respective to *S. maltophilia* K279a from GenBank, NCBI; *^c^* Percentage of BLAST identities of sequenced PCR products respective to the *S. maltophilia* genome is indicated in the bracket.

**Table 3:** Functional roles (RAST server), the sequences of the PCR primers and amplification parameters.

Targets	Abbrev. (RAST)	Primer Pairs	Target Sequence (5′–3′)	Amplicon Size (bp)	T_a_ (°C)
**Iron siderophore sensor & receptor system**					
Sigma factor ECF subfamily	FeSreg	FeSreg-F FeSreg-R	TTCATCGCGCGCTATCTC AGGATGGTCCGGGTGAT	275	50
Iron siderophore receptor protein	FeSR	FeSR-F FeSR-R	CAATCGCAGCGTACCTACC CGGCCACGTTGAAGAACT	271	51
Iron siderophore sensor protein	FeSS	FeSS-F FeSS-R	ACGTCGTGCAGAACGTAAC GGGTTTCCACCAGGTCATC	237	51
**Heme, hemin uptake and utilization systems in Gram-positives**					
Heme oxygenase, associated with heme uptake	HemO/HO	HemO-F HemO-R	CAGCAATTTCGCCCGTTTC GCTTGGCAGCCATCTTGTA	281	51
Heme ABC transporter, ATPase component	HmuV	HmuV-F HmuV-R	GAAGCTGCATGAGGTGGT TCTACGCTGAAGGCGAAAC	273	51
Hypothetical protein related to heme utilization	Hyp1	Hyp1-F Hyp1-R	GGCATCGTCGGCATCTT ACTTCACCCAGGCAATCG	247	51
Heme ABC transporter, permease protein	HmuU	HmuU-F HmuU-R	ACGCCATTGGACATGCT AACAAGCCCAGCGGAAT	299	50
Heme ABC transporter, cell surface heme and hemoprotein receptor	HmuT	HmuT-F HmuT-R	CATGCGCCACGACTGAT CATCACCCAGACCCGATTG	300	51
**Heme, hemin uptake and utilization systems in Gram-negatives**					
Outer membrane receptor proteins, mostly Fe transport	Rp2	Rp2-F Rp2-R	AACGCATGCCCGACTAC CTGGCTCATGCCCATCAT	221	51
Hemin uptake protein	Hup	Hup-F Hup-R	ATGCTCATGAATGCTCAACC TACTTGGTCAGGATCAGCTTG	200	51
Electron transfer flavoprotein, beta subunit	ETFb	ETFb-F ETFb-R	CCTGGAAACGCTGGAAGT CCTTGACCATCACACCCTT	215	51
Ferric siderophore transport system, periplasmic binding protein	TonB	TonB-F TonB-R	CGCGAGAACCGCATGTAT TCCTCGGCGTCCTTCTT	314	51
Ferric siderophore transport system, biopolymer transport protein	ExbB	ExbB-F ExbB-R	GAGCGTTTCTGGTCCCTTC CCCAGTGCGTTCAGGAAT	251	51
Hemin transport protein	Htp	Htp-F Htp-R	CACCGTGTTGTGCCTGTA GCCTCGCTATCGTGTTCC	234	51
TonB-dependent hemin, ferrichrome receptor	FCR	FCR-F FCR-R	CGGAAATGAAGGCCGGTATC CCATTCGATGTAGCGCTTGT	380	51
**Encapsulating protein DyP-type peroxidase and ferritin-like protein oligomers**					
Predicted dye-decolorizing peroxidase, encapsulated subgroup	DyP	DyP-F DyP-R	GTGCTGAAGGTGAAGGATGA TGCACGGATGTGGTACAG	258	50
**Oxidative stress related to iron uptake**					
Ferric uptake regulation protein FUR	Fur	Fur-F Fur-R	TGACCGCCGAAGACATCTA GCGAGTGCTCTTCCAGTTC	279	51

**Abbrev.**: Abbreviation; **F**: forward; **R**: reverse; **bp**: base pair; **T_a_**: annealing temperature.

## Supplementary Materials

The following are available online, Table S1: RAST subsystem information for *S. maltophilia* strain K279a, R551-3, D457 and JV3. Table S2: BLAST output for 17 functional targets. Table S3: Fold changes in expression under iron-depleted and iron-repleted conditions screened through nCounter Elements for clinical and environmental isolates. Table S4: Mean OD reading for *N. meningitidis*, SM77 and LMG10879 under the iron-depleted and iron-repleted conditions. Table S5: nCounter Elements design details for iron acquisition gene expression in *S. maltophilia*.
