# Supplementary material for: Putative Iron Acquisition Systems in Stenotrophomonas maltophilia
Source: Molecules. 2018 Aug 16;23(8):2048. doi: 10.3390/molecules23082048 (PMC6222749; doi:10.3390/molecules23082048)
Supplement: Supplementary file 1 [file molecules-23-02048-s001.zip › suppl proof/Table S1 SM IAS.pdf]

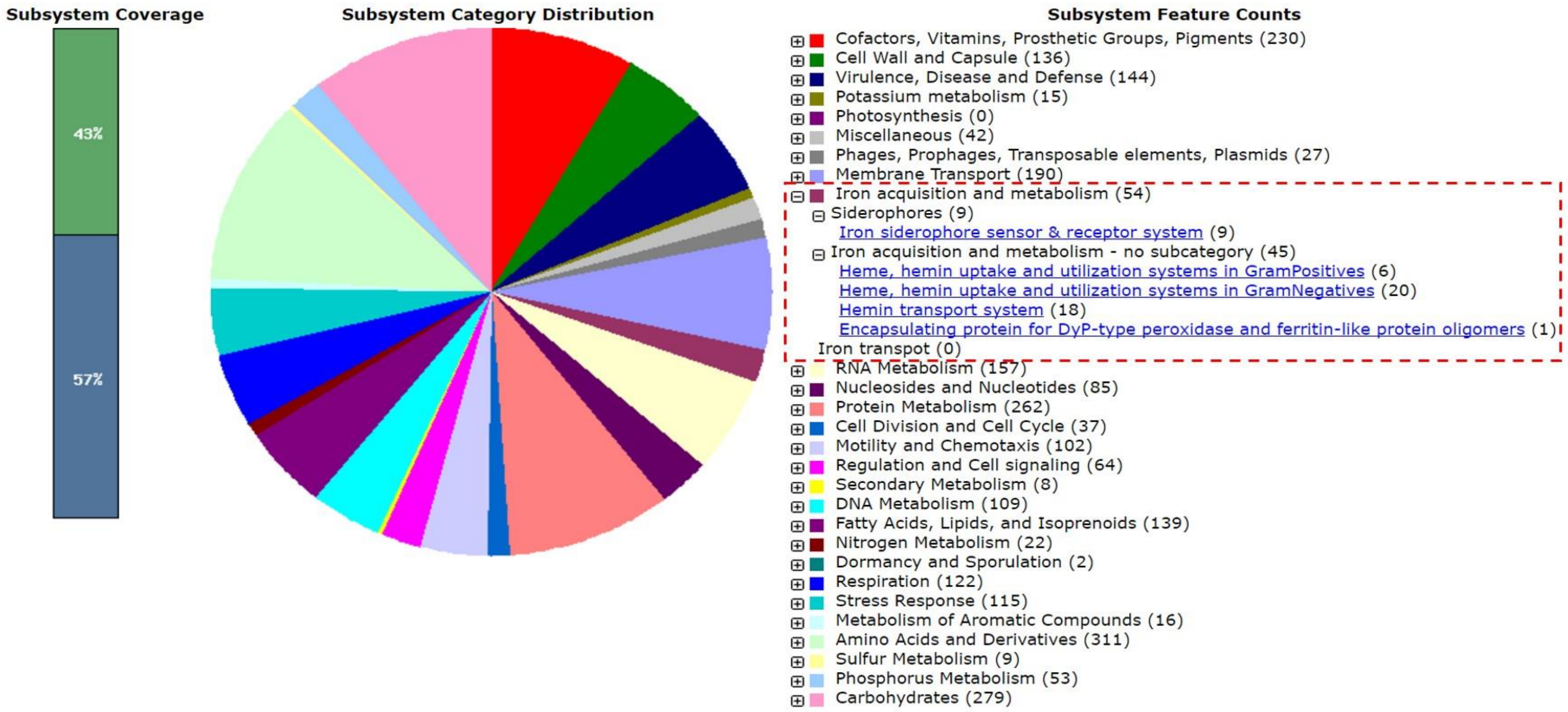

Figure S1: RAST subsystem information (subsystem statistics) for *S. maltophilia* K279a. The subsystem features count for the iron acquisition and metabolism were 54/2730 (**marked red in box**), subcategorized under siderophore (9), iron acquisition and metabolism – no subcategory (45) and iron transport (0). The functional roles were “iron siderophore sensor & receptor system (9)”; “heme, hemin uptake and utilization systems in Gram-Positives (6)”; “heme, hemin uptake and utilization systems in Gram-Negatives (20)”; “hemin transport system (18)” and “encapsulating protein for DyP-type peroxidase and ferritin-like protein oligomers (1)”.

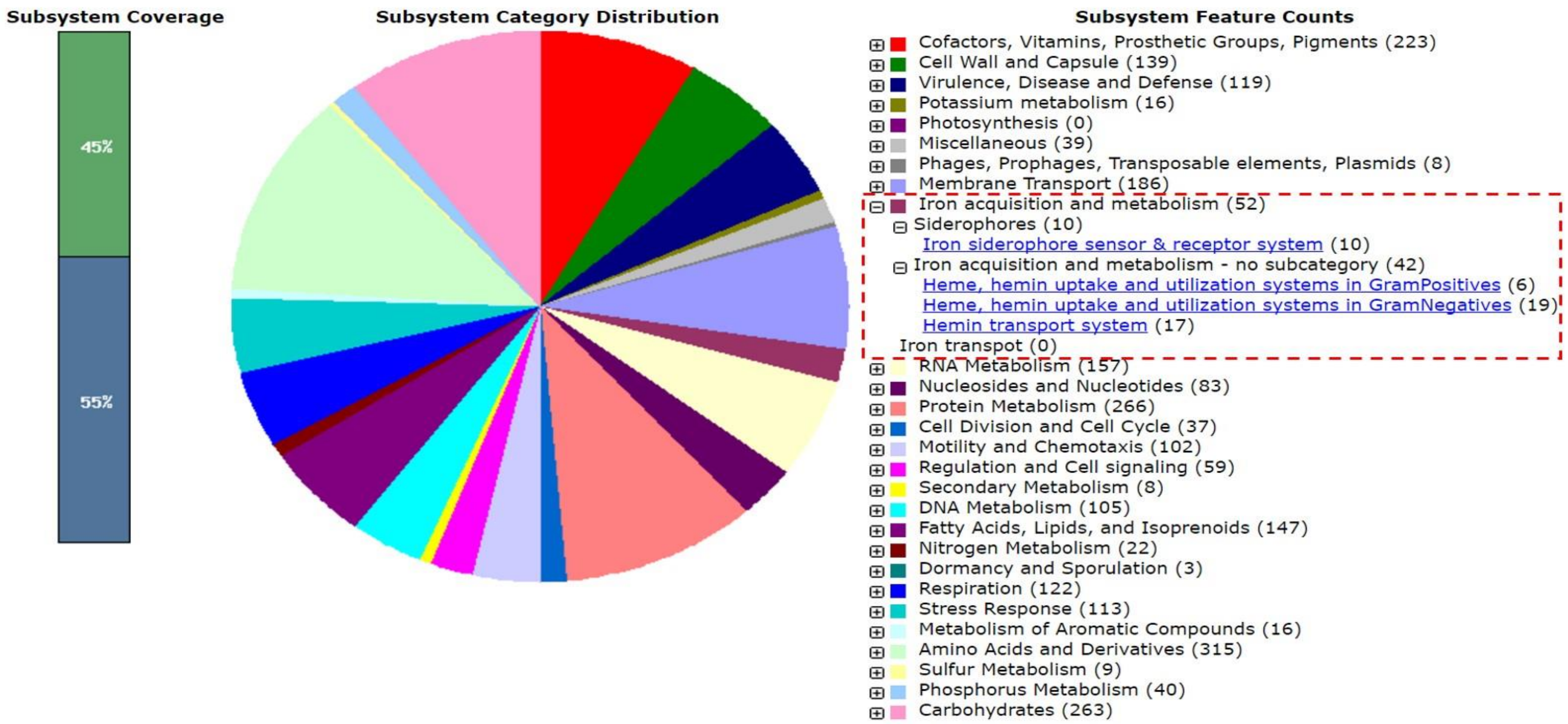

Figure S2: RAST subsystem information (subsystem statistics) for *S. maltophilia* R551-3. The subsystem features count for the iron acquisition and metabolism were 52/2649 (**marked red in box**), subcategorized under siderophore (10), iron acquisition and metabolism – no subcategory (42) and iron transport (0). The functional roles were “iron siderophore sensor & receptor system (10)”; “heme, hemin uptake and utilization systems in Gram-Positives (6)”; “heme, hemin uptake and utilization systems in Gram-Negatives (19)” and “hemin transport system (17)”.

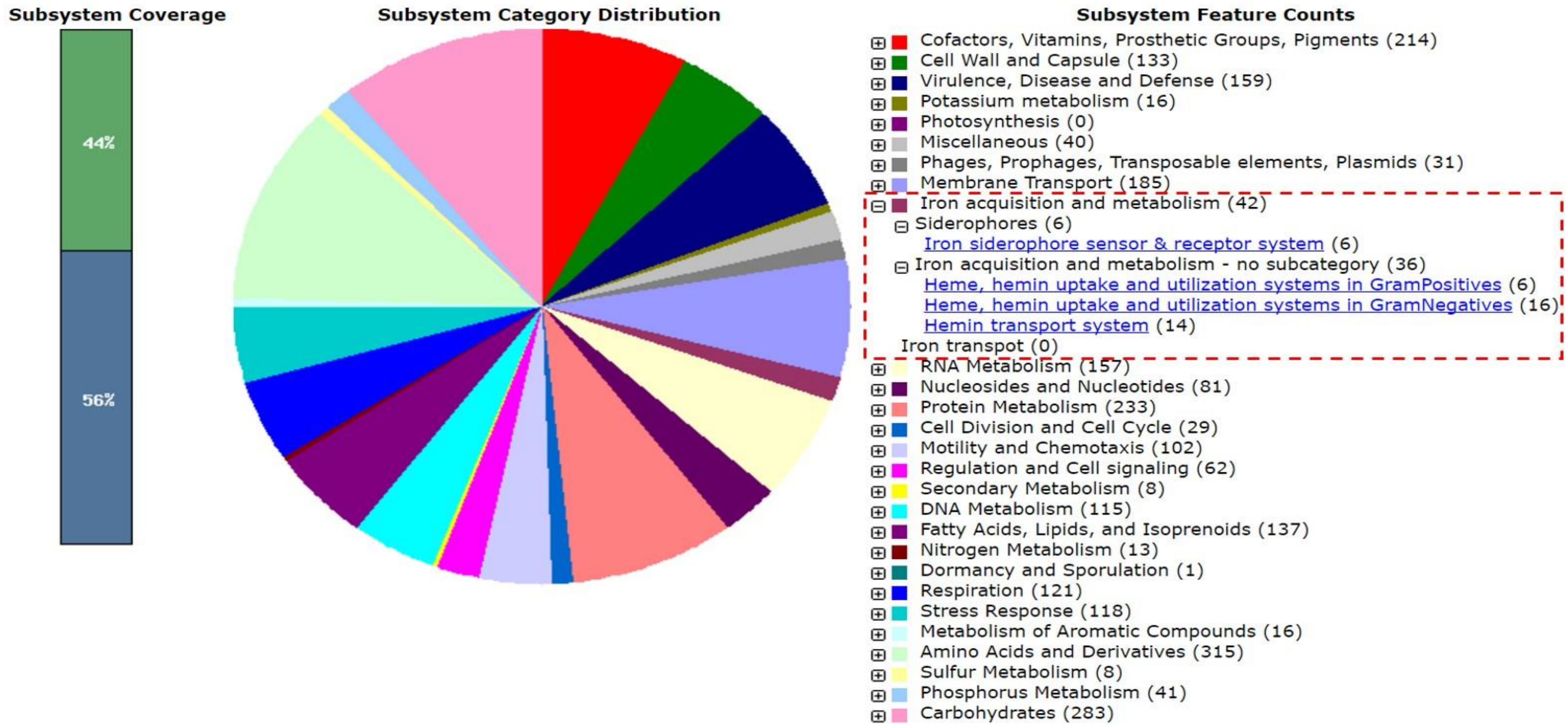

Figure S3: RAST subsystem information (subsystem statistics) for *S. maltophilia* D457. The subsystem features count for the iron acquisition and metabolism were 42/2660 (**marked red in box**), subcategorized under siderophore (6), iron acquisition and metabolism – no subcategory (36) and iron transport (0). The functional roles were "iron siderophore sensor & receptor system (6)"; "heme, hemin uptake and utilization systems in Gram-Positives (6)"; "heme, hemin uptake and utilization systems in Gram-Negatives (16)" and "hemin transport system (14)".

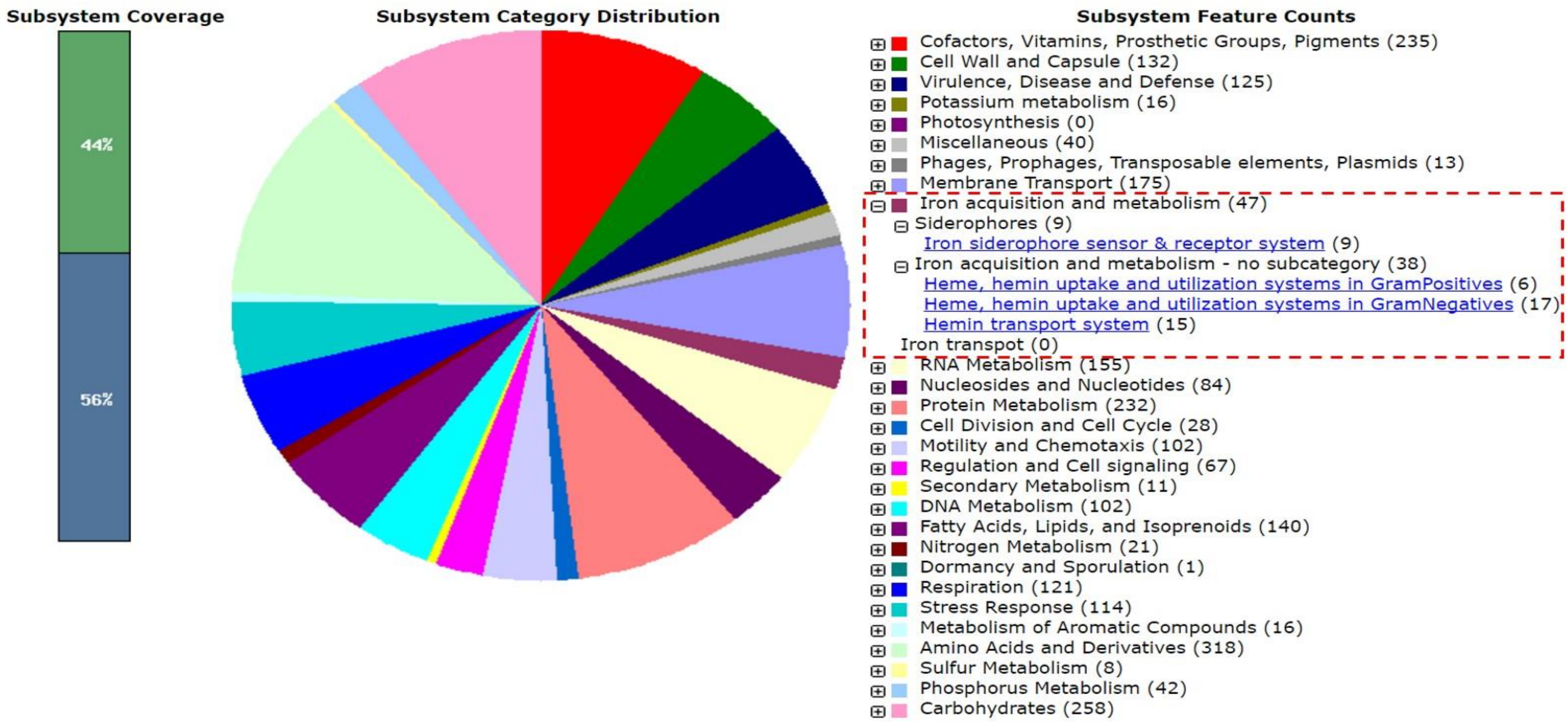

Figure S4: RAST subsystem information (subsystem statistics) for *S. maltophilia* JV3. The subsystem features count for the iron acquisition and metabolism were 47/2603 (**marked red in box**), subcategorized under siderophore (9), iron acquisition and metabolism – no subcategory (38) and iron transport (0). The functional roles were “iron siderophore sensor & receptor system (9)”; “heme,hemin uptake and utilization systems in Gram-Positives (6)”; “heme, hemin uptake and utilization systems in Gram-Negatives (17)” and “hemin transport system (15)”
