# Supplementary material for: Putative Iron Acquisition Systems in Stenotrophomonas maltophilia
Source: Molecules. 2018 Aug 16;23(8):2048. doi: 10.3390/molecules23082048 (PMC6222749; doi:10.3390/molecules23082048)
Supplement: Supplementary file 1 [file molecules-23-02048-s001.zip › suppl proof/Table S2 SM IAS.pdf]

Sigma factor ECF subfamily (FeSreg)

Description	Max score	Total score	Query cover	E value	Ident	Accession
Stenotrophomonas maltophilia K279a complete genome, strain K279a	459	459	100%	2e-125	97%	AM743169.1
Stenotrophomonas maltophilia strain 13637 genome	448	448	100%	4e-122	96%	CP008838.1
Stenotrophomonas maltophilia strain ISMMS3, complete genome	431	431	100%	4e-117	95%	CP011010.1
Stenotrophomonas maltophilia R551-3, complete genome	420	420	100%	9e-114	94%	CP001111.1
Stenotrophomonas maltophilia D457 complete genome	409	409	100%	2e-110	93%	HE798556.1
Stenotrophomonas maltophilia JV3, complete genome	403	403	100%	9e-109	93%	CP002986.1
Stenotrophomonas maltophilia strain ISMMS2, complete genome	392	392	100%	2e-105	92%	CP011305.1
Stenotrophomonas maltophilia strain ISMMS2R, complete genome	392	392	100%	2e-105	92%	CP011306.1

Stenotrophomonas maltophilia K279a complete genome, strain K279a

Sequence ID: emb|AM743169.1| **Length:** 4851126 **Number of Matches:** 1

Range 1: 2742086 to 2742360 GenBank Graphics ▼ Next Match ▲ Previous Match

Score	Expect	Identities	Gaps	Strand
459 bits(248)	2e-125	266/275(97%)	0/275(0%)	Plus/Minus

```
Query  1        TTCATCGCGCGCTATCTCGACCCGGCCAGTACCGAGGACACCCTGCAGAACCTGTACCTG  60
                ||||||||||||||||||||||||||||||||||||||||||||||||||||||||||||
Sbjct  2742360  TTCATCGCGCGCTATCTCGACCCGGCCAGTACCGAGGACACCCTGCAGAACCTGTACCTG  2742301

Query  61       AAGGCCAGCAGCGTGCCCGGCGATCCGCCCATCCTCGAGCCGCGCGGCTATCTGTACCGG  120
                ||||||||||||||||||||||||||||||||||| ||||||||||||||||||||||||
Sbjct  2742300  AAGGCCAGCAGCGTGCCCGGCGATCCGCCCATCCTGGAGCCGCGCGGCTATCTGTACCGG  2742241

Query  121      ATGGCCTACCACCACGCGCTCAACCGCAGCCAGTCCGACGCCCGCGAGCGGCGTGCGATG  180
                ||||||||||| |||||||||||||||||||||||||||||||||||||||||||| |||
Sbjct  2742240  ATGGCCTACCATCACGCGCTCAACCGCAGCCAGTCCGACGCCCGCGAGCGGCGTGCCATG  2742181

Query  181      GCCGAGTACGCCCTCGACATGGCCGAGGCCAGCCGCGATGGCGAGGCGCAGGCGCTGGAC  240
                |||||||||||||||||||||||||||||  ||| ||| |||||||||||||||||||||
Sbjct  2742180  GCCGAGTACGCCCTCGACATGGCCGAGGCGGGCCACGACGGCGAGGCGCAGGCGCTGGAC  2742121

Query  241      CAGGCGCAGCTGCGCGAGATCACCCGGACCATCCT  275
                ||||| || ||||||||||||||||||||||||||
Sbjct  2742120  CAGGCACAACTGCGCGAGATCACCCGGACCATCCT  2742086
```

Stenotrophomonas maltophilia K279a complete genome, strain K279a

GenBank: AM743169.1

```
 FEATURES             Location/Qualifiers
      source          1..275
                      /organism="Stenotrophomonas maltophilia K279a"
                      /mol_type="genomic DNA"
                      /strain="K279a"
                      /db_xref="taxon:522373"
                      /country="United Kingdom"
      gene            complement(<1..>275)
                      /locus_tag="Smlt2716"
      CDS             complement(<1..>275)
                      /locus_tag="Smlt2716"
                      /note="similarity:fasta; BB4742; bbronchiseptica; ECF
                      sigma factor; length 177 aa; id=39.8%; E()=1.7e-11; 133 aa
                      overlap; query 6-136 aa; subject 30-162 aa"
                      /codon_start=1
                      /transl_table=11
                      /product="putative ECF sigma factor"
```

Iron siderophore receptor protein (FeSR)

Description	Max score	Total score	Query cover	E value	Ident	Accession
Stenotrophomonas maltophilia D457 complete genome	484	484	98%	3e-133	99%	HE798556.1
Stenotrophomonas maltophilia JV3, complete genome	401	401	97%	3e-108	94%	CP002986.1
Stenotrophomonas maltophilia strain ISMMS2, complete genome	396	396	97%	1e-106	94%	CP011305.1
Stenotrophomonas maltophilia strain ISMMS2R, complete genome	396	396	97%	1e-106	94%	CP011306.1
Stenotrophomonas maltophilia K279a complete genome, strain K279a	390	390	100%	7e-105	93%	AM743169.1
Stenotrophomonas maltophilia strain 13637 genome	385	385	100%	3e-103	92%	CP008838.1
Stenotrophomonas maltophilia R551-3, complete genome	385	385	98%	3e-103	93%	CP001111.1
Stenotrophomonas maltophilia strain ISMMS3, complete genome	357	357	98%	7e-95	91%	CP011010.1

Stenotrophomonas maltophilia D457 complete genome

Sequence ID: emb|HE798556.1| **Length:** 4769156 **Number of Matches:** 1

Range 1: 3908959 to 3909226 GenBank Graphics ▼ Next Match ▲ Previous Match

Score	Expect	Identities	Gaps	Strand
484 bits(262)	3e-133	266/268(99%)	0/268(0%)	Plus/Minus

```
Query  4        TCGCAGCGTACCTACCTCGACGCGGACCTGATCTCCAGCCTGACCATCACCAAGGGCCCG  63
                |||||||| |||||||||||| ||||||||||||||||||||||||||||||||||||||
Sbjct  3909226  TCGCAGCGCACCTACCTCGACCCGGACCTGATCTCCAGCCTGACCATCACCAAGGGCCCG  3909167

Query  64       AGCCTGCAGGCCAACGCGTCCGGTGGCATCGGCGGCGTGGTCGAGATGGAGACGTTGAAG  123
                ||||||||||||||||||||||||||||||||||||||||||||||||||||||||||||
Sbjct  3909166  AGCCTGCAGGCCAACGCGTCCGGTGGCATCGGCGGCGTGGTCGAGATGGAGACGTTGAAG  3909107

Query  124      ATCGGTGACGTGCTGCGCGAGGGCCGCGACGTCGGCGTGCGCGTGCGCGGCGGCCTGGCC  183
                ||||||||||||||||||||||||||||||||||||||||||||||||||||||||||||
Sbjct  3909106  ATCGGTGACGTGCTGCGCGAGGGCCGCGACGTCGGCGTGCGCGTGCGCGGCGGCCTGGCC  3909047

Query  184      AATGGCAGTGCCAACAGCCTGCCGTCCTACAGTGCGGCACCGCGCACCGATCGCAGCGCC  243
                ||||||||||||||||||||||||||||||||||||||||||||||||||||||||||||
Sbjct  3909046  AATGGCAGTGCCAACAGCCTGCCGTCCTACAGTGCGGCACCGCGCACCGATCGCAGCGCC  3908987

Query  244      ACCGGCAGCCAGTTCTTCAACGTGGCCG  271
                ||||||||||||||||||||||||||||
Sbjct  3908986  ACCGGCAGCCAGTTCTTCAACGTGGCCG  3908959
```

Stenotrophomonas maltophilia D457 complete genome

GenBank: HE798556.1

```
FEATURES             Location/Qualifiers
     source          1..268
                     /organism="Stenotrophomonas maltophilia D457"
                     /mol_type="genomic DNA"
                     /strain="D457"
                     /db_xref="taxon:1163399"
                     /country="Spain:Mostoles"
     gene            complement(<1..>268)
                     /locus_tag="SMD_3500"
     CDS             complement(<1..>268)
                     /locus_tag="SMD_3500"
                     /codon_start=1
                     /transl_table=11
                     /product="Iron siderophore receptor protein"
```

Iron siderophore sensor protein (FeSS)

Description	Max score	Total score	Query cover	E value	Ident	Accession
Stenotrophomonas maltophilia K279a complete genome, strain K279a	427	427	100%	4e-116	99%	AM743169.1
Stenotrophomonas maltophilia strain 13637 genome	411	411	100%	4e-111	98%	CP008838.1
Stenotrophomonas maltophilia strain ISMMS2, complete genome	311	311	100%	5e-81	90%	CP011305.1
Stenotrophomonas maltophilia strain ISMMS2R, complete genome	311	311	100%	5e-81	90%	CP011306.1
Stenotrophomonas maltophilia R551-3, complete genome	285	285	99%	3e-73	89%	CP001111.1
Stenotrophomonas maltophilia strain ISMMS3, complete genome	272	272	100%	2e-69	87%	CP011010.1

Stenotrophomonas maltophilia K279a complete genome, strain K279a

Sequence ID: emb|AM743169.1| **Length:** 4851126 **Number of Matches:** 1

Range 1: 3988174 to 3988410 GenBank Graphics ▼ Next Match ▲ Previous Match

Score	Expect	Identities	Gaps	Strand
427 bits(231)	4e-116	235/237(99%)	0/237(0%)	Plus/Minus

```
Query  1        ACGTCGTGCAGAACGTAACGCTGCCCGATGGCAGCCGCGCAGTGCTGGATGCGGACAGCG  60
                ||||||||||||||||||||||||||||||||||||||||||||||||||||||||||||
Sbjct  3988410  ACGTCGTGCAGAACGTAACGCTGCCCGATGGCAGCCGCGCAGTGCTGGATGCGGACAGCG  3988351

Query  61       CGATCGCCGTGCGCTTCGATGCGCACGCACGCGAGGTTGAGCTCCTGCGCGGGCGCGTCT  120
                ||||||||||||||||||||||||||||||||||||| ||||| ||||||||||||||||
Sbjct  3988350  CGATCGCCGTGCGCTTCGATGCGCACGCACGCGAGGTCGAGCTGCTGCGCGGGCGCGTCT  3988291

Query  121      GGTTCGAGGTCAGCCCGGATGCGCAGCGGCGTTTCAGCGTGCGTGCCGGCAATGGCGTGG  180
                ||||||||||||||||||||||||||||||||||||||||||||||||||||||||||||
Sbjct  3988290  GGTTCGAGGTCAGCCCGGATGCGCAGCGGCGTTTCAGCGTGCGTGCCGGCAATGGCGTGG  3988231

Query  181      TTGAGGACATCTCGACCGCGTTCACCGTCGCGCGTGGCGATGACCTGGTGGAAACCC  237
                |||||||||||||||||||||||||||||||||||||||||||||||||||||||||
Sbjct  3988230  TTGAGGACATCTCGACCGCGTTCACCGTCGCGCGTGGCGATGACCTGGTGGAAACCC  3988174
```

Stenotrophomonas maltophilia K279a complete genome, strain K279a

GenBank: AM743169.1

```
FEATURES             Location/Qualifiers
     source          1..237
                     /organism="Stenotrophomonas maltophilia K279a"
                     /mol_type="genomic DNA"
                     /strain="K279a"
                     /db_xref="taxon:522373"
                     /country="United Kingdom"
     gene            complement(<1..>237)
                     /locus_tag="Smlt3899"
     CDS             complement(<1..>237)
                     /locus_tag="Smlt3899"
                     /note="similarity:fasta; ECA1539; ecarotovora_atroseptica;
                     putative transmembrane sensor protein; length 322 aa;
                     id=31.5%; E()=3.9e-20; 317 aa overlap; query 25-318 aa;
                     subject 10-322 aa;
                     similarity:fasta; with=UniProt:Q1IGG4; Pseudomonas
                     entomophila (strain L48).; Putative FecR-like
                     transmembrane sensor.; length=325; id 33.974%; ungapped id
                     36.426%; E()=8.8e-21; 312 aa overlap; query 9-306; subject
                     7-311"
                     /codon_start=1
                     /transl_table=11
                     /product="putative transmembrane FecR sensor protein"
```

Heme oxygenase, associated with heme uptake (HemO/HO)

Description	Max score	Total score	Query cover	E value	Ident	Accession
Stenotrophomonas maltophilia strain 13637 genome	520	520	100%	8e-144	100%	CP008838.1
Stenotrophomonas maltophilia K279a complete genome, strain K279a	514	514	100%	4e-142	99%	AM743169.1
Stenotrophomonas maltophilia R551-3, complete genome	436	436	100%	9e-119	95%	CP001111.1
Stenotrophomonas maltophilia D457 complete genome	431	431	100%	4e-117	94%	HE798556.1
Stenotrophomonas maltophilia JV3, complete genome	431	431	100%	4e-117	94%	CP002986.1
Stenotrophomonas maltophilia strain ISMMS2, complete genome	364	364	100%	4e-97	90%	CP011305.1
Stenotrophomonas maltophilia strain ISMMS2R, complete genome	364	364	100%	4e-97	90%	CP011306.1
Stenotrophomonas maltophilia strain ISMMS3, complete genome	353	353	100%	9e-94	89%	CP011010.1

Stenotrophomonas maltophilia strain 13637 genome

Sequence ID: gb|CP008838.1| **Length:** 4989312 **Number of Matches:** 1

Range 1: 4804541 to 4804821 GenBank Graphics ▼ Next Match ▲ Previous Match

Score	Expect	Identities	Gaps	Strand
520 bits(281)	8e-144	281/281(100%)	0/281(0%)	Plus/Plus

```
Query  1        CAGCAATTTCGCCCGTTTCCTGCGCGTGCAGTATCGCTTCCACCGCAGCATCGATGCGCT  60
                ||||||||||||||||||||||||||||||||||||||||||||||||||||||||||||
Sbjct  4804541  CAGCAATTTCGCCCGTTTCCTGCGCGTGCAGTATCGCTTCCACCGCAGCATCGATGCGCT  4804600

Query  61       GTATGCCAACCCTGCGCTGGACGCACTGCTGCCGGACCTGGGCGAGCGCCGCCGCCTGAC  120
                ||||||||||||||||||||||||||||||||||||||||||||||||||||||||||||
Sbjct  4804601  GTATGCCAACCCTGCGCTGGACGCACTGCTGCCGGACCTGGGCGAGCGCCGCCGCCTGAC  4804660

Query  121      CCAGGTCGCGCGCGACCTGCAGGACCTGGAGCAGACCCTGCCGGGGGCTGACATCGCGGC  180
                ||||||||||||||||||||||||||||||||||||||||||||||||||||||||||||
Sbjct  4804661  CCAGGTCGCGCGCGACCTGCAGGACCTGGAGCAGACCCTGCCGGGGGCTGACATCGCGGC  4804720

Query  181      GTTGCCGTCCGATCTGGAACTGCCCGCCGCGTTGGGGTGGCTGTACGTGGCTGAAGGCTC  240
                ||||||||||||||||||||||||||||||||||||||||||||||||||||||||||||
Sbjct  4804721  GTTGCCGTCCGATCTGGAACTGCCCGCCGCGTTGGGGTGGCTGTACGTGGCTGAAGGCTC  4804780

Query  241      CAACCTGGGCGGCACCATTCTGTACAAGATGGCTGCCAAGC  281
                |||||||||||||||||||||||||||||||||||||||||
Sbjct  4804781  CAACCTGGGCGGCACCATTCTGTACAAGATGGCTGCCAAGC  4804821
```

Stenotrophomonas maltophilia strain 13637 genome

GenBank: CP008838.1

```
FEATURES             Location/Qualifiers
     source          1..281
                     /organism="Stenotrophomonas maltophilia"
                     /mol_type="genomic DNA"
                     /strain="13637"
                     /db_xref="taxon:40324"
     gene            <1..>281
                     /locus_tag="DP16_4486"
     CDS             <1..>281
                     /locus_tag="DP16_4486"
                     /codon_start=1
                     /transl_table=11
                     /product="heme oxygenase family protein"
```

Heme ABC transporter, ATPase component (HmuV)

Description	Max score	Total score	Query cover	E value	Ident	Accession
Stenotrophomonas maltophilia K279a complete genome, strain K279a	483	483	100%	1e-132	99%	AM743169.1
Stenotrophomonas maltophilia strain 13637 genome	477	477	100%	5e-131	98%	CP008838.1
Stenotrophomonas maltophilia D457 complete genome	383	383	98%	1e-102	92%	HE798556.1
Stenotrophomonas maltophilia strain ISMMS2, complete genome	364	364	97%	4e-97	91%	CP011305.1
Stenotrophomonas maltophilia strain ISMMS2R, complete genome	364	364	97%	4e-97	91%	CP011306.1
Stenotrophomonas maltophilia strain ISMMS3, complete genome	339	339	98%	2e-89	89%	CP011010.1
Stenotrophomonas maltophilia JV3, complete genome	327	327	98%	5e-86	89%	CP002986.1

Stenotrophomonas maltophilia K279a complete genome, strain K279a

Sequence ID: emb|AM743169.1| **Length:** 4851126 **Number of Matches:** 1

Range 1: 2400245 to 2400517 GenBank Graphics ▼ Next Match ▲ Previous Match

Score	Expect	Identities	Gaps	Strand
483 bits(261)	1e-132	269/273(99%)	0/273(0%)	Plus/Plus

```
Query  1        GAAGCTGCATGAGGTGGTGGTGCGCCGCCAGCAGCGCGAGATCCTGCATGGCATCTCGCT  60
                ||||||||||||||||||||||||||||||||||||||||||||||||||||||||||||
Sbjct  2400245  GAAGCTGCATGAGGTGGTGGTGCGCCGCCAGCAGCGCGAGATCCTGCATGGCATCTCGCT  2400304

Query  61       CGCGTTCGAGCCGGGTACCGTGATCGCGCTGGTTGGCCCGAACGGCGCAGGCAAGTCCAC  120
                |||||||||||||||||| |||| ||||||||||||||||||||||||||||||||||||
Sbjct  2400305  CGCGTTCGAGCCGGGTACGGTGACCGCGCTGGTTGGCCCGAACGGCGCAGGCAAGTCCAC  2400364

Query  121      CTTGCTGGCGGTAGCGGCCGGCGACCTGCGCGCCGATGCGGGCGAGGTGAGCCTGTTGGG  180
                |||||||||| |||||||||||||||||||||||||||||||||||||||||||||||||
Sbjct  2400365  CTTGCTGGCGATAGCGGCCGGCGACCTGCGCGCCGATGCGGGCGAGGTGAGCCTGTTGGG  2400424

Query  181      CAAGCCGCTGGCCAGCTACAAGGCCGGGCCGCTGGCGCGCGAGCGCGCGGTAATGCCGCA  240
                ||| ||||||||||||||||||||||||||||||||||||||||||||||||||||||||
Sbjct  2400425  CAAACCGCTGGCCAGCTACAAGGCCGGGCCGCTGGCGCGCGAGCGCGCGGTAATGCCGCA  2400484

Query  241      GGAGCACGGCGTTCGTTTCGCCTTCAGCGTAGA  273
                |||||||||||||||||||||||||||||||||
Sbjct  2400485  GGAGCACGGCGTTCGTTTCGCCTTCAGCGTAGA  2400517
```

Stenotrophomonas maltophilia K279a complete genome, strain K279a

GenBank: AM743169.1

```
FEATURES             Location/Qualifiers
     source          1..273
                     /organism="Stenotrophomonas maltophilia K279a"
                     /mol_type="genomic DNA"
                     /strain="K279a"
                     /db_xref="taxon:522373"
                     /country="United Kingdom"
     gene            <1..>273
                     /locus_tag="Smlt2357"
     CDS             <1..>273
                     /locus_tag="Smlt2357"
                     /codon_start=1
                     /transl_table=11
                     /product="putative ABC transport protein, ATP-binding
                     component."
```

Hypothetical protein related to heme utilization (Hyp1)

Description	Max score	Total score	Query cover	E value	Ident	Accession
Stenotrophomonas maltophilia strain 13637 genome	424	424	100%	6e-115	98%	CP008838.1
Stenotrophomonas maltophilia K279a complete genome, strain K279a	424	424	100%	6e-115	98%	AM743169.1
Stenotrophomonas maltophilia strain ISMMS2, complete genome	379	379	100%	1e-101	94%	CP011305.1
Stenotrophomonas maltophilia strain ISMMS2R, complete genome	379	379	100%	1e-101	94%	CP011306.1
Stenotrophomonas maltophilia D457 complete genome	379	379	100%	1e-101	94%	HE798556.1
Stenotrophomonas maltophilia R551-3, complete genome	379	379	100%	1e-101	94%	CP001111.1
Stenotrophomonas maltophilia JV3, complete genome	368	368	100%	3e-98	94%	CP002986.1
Stenotrophomonas maltophilia strain ISMMS3, complete genome	357	357	100%	6e-95	93%	CP011010.1

Stenotrophomonas maltophilia strain 13637 genome

Sequence ID: gb|CP008838.1| **Length:** 4989312 **Number of Matches:** 1

Range 1: 4601859 to 4602105 GenBank Graphics ▼ Next Match ▲ Previous Match

Score	Expect	Identities	Gaps	Strand
424 bits(229)	6e-115	241/247(98%)	0/247(0%)	Plus/Plus

```
Query  1        GGCATCGTCGGCATCTTCGTGCCGGGCCTGCCGACCACCGTATTCATCCTGATTTCGGCC  60
                |||||||||||||||||||| |||||||||||||||||||||||||||||||||||||||
Sbjct  4601859  GGCATCGTCGGCATCTTCGTACCGGGCCTGCCGACCACCGTATTCATCCTGATTTCGGCC  4601918

Query  61       TGGGCCGCCTCGCGTGGCTCCGAGCGCCTGCACAACTGGCTGCTGCAGCATCCACGCTTC  120
                ||||||||||||||||||||||| ||||||||||||||||||||||||||||||||||||
Sbjct  4601919  TGGGCCGCCTCGCGTGGCTCCGAACGCCTGCACAACTGGCTGCTGCAGCATCCACGCTTC  4601978

Query  121      GGCCCGGCCATCGCCAACTGGCAGGCACACGGGGCGGTCAGCCGCTATGGCAAGTGGATG  180
                ||||||||||||||||||||||||||||||||||||||||||||||| ||||||||||||
Sbjct  4601979  GGCCCGGCCATCGCCAACTGGCAGGCACACGGGGCGGTCAGCCGCTACGGCAAGTGGATG  4602038

Query  181      GCCACGATCACCATGGCGGTATGCGCCGCCATCATGCTGTGGTGCGTACCGATTGCCTGG  240
                |||||||||||||||||||||||||||| ||||||||| |||||||| ||||||||||||
Sbjct  4602039  GCCACGATCACCATGGCGGTATGCGCCGGCATCATGCTCTGGTGCGTGCCGATTGCCTGG  4602098

Query  241      GTGAAGT  247
                |||||||
Sbjct  4602099  GTGAAGT  4602105
```

Stenotrophomonas maltophilia strain 13637 genome

GenBank: CP008838.1

```
FEATURES             Location/Qualifiers
     source          1..247
                     /organism="Stenotrophomonas maltophilia"
                     /mol_type="genomic DNA"
                     /strain="13637"
                     /db_xref="taxon:40324"
     gene            <1..>247
                     /locus_tag="DP16_4312"
     CDS             <1..>247
                     /locus_tag="DP16_4312"
                     /note="conserved hypothetical protein"
                     /codon_start=1
                     /transl_table=11
                     /product="hypothetical protein"
```

Heme ABC transporter, permease protein (HmuU)

Description	Max score	Total score	Query cover	E value	Ident	Accession
Stenotrophomonas maltophilia K279a complete genome, strain K279a	420	420	100%	1e-113	92%	AM743169.1
Stenotrophomonas maltophilia strain ISMMS3, complete genome	403	403	100%	1e-108	91%	CP011010.1
Stenotrophomonas maltophilia D457 complete genome	398	398	100%	4e-107	91%	HE798556.1
Stenotrophomonas maltophilia R551-3, complete genome	398	398	100%	4e-107	91%	CP001111.1
Stenotrophomonas maltophilia JV3, complete genome	381	381	100%	4e-102	90%	CP002986.1
Stenotrophomonas maltophilia strain ISMMS2, complete genome	375	375	100%	2e-100	89%	CP011305.1
Stenotrophomonas maltophilia strain ISMMS2R, complete genome	375	375	100%	2e-100	89%	CP011306.1

Stenotrophomonas maltophilia K279a complete genome, strain K279a

Sequence ID: emb|AM743169.1| **Length:** 4851126 **Number of Matches:** 1

Range 1: 2399866 to 2400164 GenBank Graphics ▼ Next Match ▲ Previous Match

Score	Expect	Identities	Gaps	Strand
420 bits(227)	1e-113	275/299(92%)	0/299(0%)	Plus/Plus

```
Query  1        ACGCCATTGGACATGCTGGCGCTGGGCGAGCGCCAGGCCCAGCACCTGGGGCTGGATGTG  60
                ||||||||||||||||||||||||||||| |||||||| |||||||| |||||||| |||
Sbjct  2399866  ACGCCATTGGACATGCTGGCGCTGGGCGAACGCCAGGCGCAGCACCTCGGGCTGGACGTG  2399925

Query  61       ACCCGCACGCGGCGTCGACTGGTGGCGTTCAGCGCGCTGCTGGTGGGGGCGGCGGTGGCG  120
                ||||||||||||||||| |||||||||||||| |||||||||||||| ||||||||||| 
Sbjct  2399926  ACCCGCACGCGGCGTCGCCTGGTGGCGTTCAGTGCGCTGCTGGTGGGCGCGGCGGTGGCC  2399985

Query  121      TTTGCGGGCTCGATCAGCTTCGTTGGCCTGGTGGTGCCGCACGTGGCGCGCCTGCTGGTG  180
                ||||| || |||||||| |||||||||||||| |||||||||||||||||||||||||| 
Sbjct  2399986  TTTGCCGGTTCGATCAGTTTCGTTGGCCTGGTAGTGCCGCACGTGGCGCGCCTGCTGGTC  2400045

Query  181      GGTCCCGGTCATCGCTGGCTGCTGCCGTTGTCCGGCCTGCTGGGTGCACTGCTGATCGTG  240
                || ||||| ||||||||||||||||||||||||||||||||||| || ||||||||||||
Sbjct  2400046  GGCCCCGGGCATCGCTGGCTGCTGCCGTTGTCCGGCCTGCTGGGCGCGCTGCTGATCGTG  2400105

Query  241      GTGGCGGATACGGCCGCGCGCACGCTGGATCCGCCCGCGGAGATTCCGCTGGGCTTGTT  299
                ||||| || || || || |||||||| |||||||| |||||||||||||||||||||||
Sbjct  2400106  GTGGCCGACACCGCGGCCCGCACGCTCGATCCGCCGGCGGAGATTCCGCTGGGCTTGTT  2400164
```

Stenotrophomonas maltophilia K279a complete genome, strain K279a

GenBank: AM743169.1

```
FEATURES             Location/Qualifiers
     source          1..299
                     /organism="Stenotrophomonas maltophilia K279a"
                     /mol_type="genomic DNA"
                     /strain="K279a"
                     /db_xref="taxon:522373"
                     /country="United Kingdom"
     gene            <1..>299
                     /locus_tag="Smlt2356"
     CDS             <1..>299
                     /locus_tag="Smlt2356"
                     /codon_start=1
                     /transl_table=11
                     /product="putative FecCD-family transmembrane transport
                     protein."
```

Heme ABC transporter, cell surface heme and hemoprotein receptor (HmuT)

Description	Max score	Total score	Query cover	E value	Ident	Accession
Stenotrophomonas maltophilia D457 complete genome	499	499	100%	1e-137	97%	HE798556.1
Stenotrophomonas maltophilia strain 13637 genome	455	455	100%	3e-124	94%	CP008838.1
Stenotrophomonas maltophilia K279a complete genome, strain K279a	455	455	100%	3e-124	94%	AM743169.1
Stenotrophomonas maltophilia strain ISMMS2, complete genome	444	444	100%	6e-121	93%	CP011305.1
Stenotrophomonas maltophilia strain ISMMS2R, complete genome	444	444	100%	6e-121	93%	CP011306.1
Stenotrophomonas maltophilia JV3, complete genome	444	444	100%	6e-121	93%	CP002986.1
Stenotrophomonas maltophilia R551-3, complete genome	427	427	100%	6e-116	92%	CP001111.1
Stenotrophomonas maltophilia strain ISMMS3, complete genome	399	399	100%	1e-107	91%	CP011010.1

Stenotrophomonas maltophilia D457 complete genome

Sequence ID: emb|HE798556.1| **Length:** 4769156 **Number of Matches:** 1

Range 1: 2356617 to 2356916 GenBank Graphics ▼ Next Match ▲ Previous Match

Score	Expect	Identities	Gaps	Strand
499 bits(270)	1e-137	290/300(97%)	0/300(0%)	Plus/Plus

```
Query  1        CATGCGCCACGACTGATCCACGTCTCGGCAACCGGCGCTGGCGGCTCGCCCACTGTTGCC  60
                ||||||||||||||||||||||| ||||| ||||||||||||||||||||||||||||||
Sbjct  2356617  CATGCGCCACGACTGATCCACGTATCGGCCACCGGCGCTGGCGGCTCGCCCACTGTTGCC  2356676

Query  61       GGTGCCGACAGCGCGTCGGCGCAGCTGATCGCCCTGGCCGGCGGCATCAACATCGGCACC  120
                ||||||||||||||||| ||||||||||||||| ||||||||||||||||||||||||||
Sbjct  2356677  GGTGCCGACAGCGCGTCAGCGCAGCTGATCGCGCTGGCCGGCGGCATCAACATCGGCACC  2356736

Query  121      GAGGCCGGGGTGAAGAACTACTCGCAGCTGAGCAACGAGGGCGTGGTCGCGGCGGCGCCG  180
                ||||||||||||||||||||||||||||||||||||||||| |||||||| ||||| |||
Sbjct  2356737  GAGGCCGGGGTGAAGAACTACTCGCAGCTGAGCAACGAGGGGGTGGTCGCCGCGGCACCG  2356796

Query  181      GAAGTGATCCTGGTGACCGAGCATGACCTGCAGCTGTTCGGCGGTGCCGAAGGCCTGTGG  240
                || |||||||||||||||||||||||||||| ||||||||||||||||||||||||||||
Sbjct  2356797  GAGGTGATCCTGGTGACCGAGCATGACCTGCGGCTGTTCGGCGGTGCCGAAGGCCTGTGG  2356856

Query  241      AAGGCGTACCCGACGCTGAAGCAGACCCCGGCCGGGCAGGCCAATCGGGTCTGGGTGATG  300
                |||||||||||||||||||||||||| |||||||||||||||||||||||||||||||||
Sbjct  2356857  AAGGCGTACCCGACGCTGAAGCAGACGCCGGCCGGGCAGGCCAATCGGGTCTGGGTGATG  2356916
```

Stenotrophomonas maltophilia D457 complete genome

GenBank: HE798556.1

```
FEATURES             Location/Qualifiers
     source          1..300
                     /organism="Stenotrophomonas maltophilia D457"
                     /mol_type="genomic DNA"
                     /strain="D457"
                     /db_xref="taxon:1163399"
                     /country="Spain:Mostoles"
     gene            <1..>300
                     /locus_tag="SMD_2121"
     CDS             <1..>300
                     /locus_tag="SMD_2121"
                     /codon_start=1
                     /transl_table=11
                     /product="Heme ABC transporter, cell surface heme and
                     hemoprotein receptor HmuT"
```

Outer membrane receptor proteins, mostly Fe transport (Rp2)

Description	Max score	Total score	Query cover	E value	Ident	Accession
Stenotrophomonas maltophilia JV3, complete genome	348	348	100%	3e-92	95%	CP002986.1
Stenotrophomonas maltophilia D457 complete genome	342	342	100%	1e-90	95%	HE798556.1
Stenotrophomonas maltophilia strain ISMMS2, complete genome	327	327	99%	4e-86	94%	CP011305.1
Stenotrophomonas maltophilia strain ISMMS2R, complete genome	327	327	99%	4e-86	94%	CP011306.1
Stenotrophomonas maltophilia strain ISMMS3, complete genome	327	327	99%	4e-86	94%	CP011010.1
Stenotrophomonas maltophilia strain 13637 genome	315	315	100%	3e-82	92%	CP008838.1
Stenotrophomonas maltophilia K279a complete genome, strain K279a	315	315	100%	3e-82	92%	AM743169.1
Stenotrophomonas maltophilia R551-3, complete genome	278	278	99%	4e-71	89%	CP001111.1

Stenotrophomonas maltophilia D457 complete genome

Sequence ID: emb|HE798556.1| **Length:** 4769156 **Number of Matches:** 1

Range 1: 3781688 to 3781908 GenBank Graphics ▼ Next Match ▲ Previous Match

Score	Expect	Identities	Gaps	Strand
342 bits(185)	1e-90	209/221(95%)	0/221(0%)	Plus/Minus

```
Query  1        AACGCATGCCCGACTACTGGGAGTTGTTCTCGCCCGACCACGGCCCGGCCGGTGCGGTGA  60
                ||||||||||||||||||||||| ||||||||||||||||||||||||||| ||||||||
Sbjct  3781908  AACGCATGCCCGACTACTGGGAGCTGTTCTCGCCCGACCACGGCCCGGCCGATGCGGTGA  3781849

Query  61       ACGCGTTTGCCGGCATCCAGCCCGAGCGCACCACCCAGCTCGACGTCGGCCTGCAGTACA  120
                |||||||||||||||||||||||||||||||||||||||||||||  |||||||||||||
Sbjct  3781848  ACGCGTTTGCCGGCATCCAGCCCGAGCGCACCACCCAGCTCGACGCTGGCCTGCAGTACA  3781789

Query  121      AGGGACGACGCGTGCAGGCCTGGGTCTCGGCCTACGCCGGGCAGATCCAGGACTACATCC  180
                ||||||  ||||||||||||||||||||||| ||| ||||||||||||||||||||||||
Sbjct  3781788  AGGGACCGCGCGTGCAGGCCTGGGTCTCGGCGTACACCGGGCAGATCCAGGACTACATCC  3781729

Query  181      TGTTCACCTACCACGGCAGCGGCATGATGGGCATGAGCCAG  221
                |||||||||||||| ||||||||  |||||| |||||||||
Sbjct  3781728  TGTTCACCTACCACCGCAGCGGCGCGATGGGGATGAGCCAG  3781688
```

Stenotrophomonas maltophilia D457 complete genome

GenBank: HE798556.1

```
FEATURES             Location/Qualifiers
     source          1..221
                     /organism="Stenotrophomonas maltophilia D457"
                     /mol_type="genomic DNA"
                     /strain="D457"
                     /db_xref="taxon:1163399"
                     /country="Spain:Mostoles"
     gene            complement(<1..>221)
                     /locus_tag="SMD_3393"
     CDS             complement(<1..>221)
                     /locus_tag="SMD_3393"
                     /codon_start=1
                     /transl_table=11
                     /product="Outer membrane receptor proteins, mostly Fe
                     transport"
```

Hemin uptake protein (Hup)

Description	Max score	Total score	Query cover	E value	Ident	Accession
Brugia timori genome assembly B_timori_Indonesia_v1_0_4, scaffold BTMF_contig0011954	370	370	100%	6e-99	100%	LK924066.1
Stenotrophomonas maltophilia D457 complete genome	370	370	100%	6e-99	100%	HE798556.1
Stenotrophomonas maltophilia strain ISMMS3, complete genome	364	364	100%	3e-97	99%	CP011010.1
Stenotrophomonas maltophilia JV3, complete genome	359	359	100%	1e-95	99%	CP002986.1
Stenotrophomonas maltophilia R551-3, complete genome	359	359	100%	1e-95	99%	CP001111.1
Stenotrophomonas maltophilia K279a complete genome, strain K279a	359	359	100%	1e-95	99%	AM743169.1
Stenotrophomonas maltophilia strain ISMMS2, complete genome	353	353	100%	6e-94	99%	CP011305.1
Stenotrophomonas maltophilia strain ISMMS2R, complete genome	353	353	100%	6e-94	99%	CP011306.1
Stenotrophomonas maltophilia strain 13637 genome	353	353	100%	6e-94	99%	CP008838.1

Stenotrophomonas maltophilia D457 complete genome

Sequence ID: emb|HE798556.1| **Length:** 4769156 **Number of Matches:** 1

Range 1: 784415 to 784614 GenBank Graphics ▼ Next Match ▲ Previous Match

Score	Expect	Identities	Gaps	Strand
370 bits(200)	6e-99	200/200(100%)	0/200(0%)	Plus/Plus

```
Query  1       ATGCTCATGAATGCTCAACCTGTACTGCTGCGCCCCGAAACGCTGACCCTCCGCGATCGT  60
               ||||||||||||||||||||||||||||||||||||||||||||||||||||||||||||
Sbjct  784415  ATGCTCATGAATGCTCAACCTGTACTGCTGCGCCCCGAAACGCTGACCCTCCGCGATCGT  784474

Query  61      CCGGTCCGTGTCGTTCCGCCGGAAGAGGTCATCGACAGCGAAGCCCTGCTCAAGGGCCGT  120
               ||||||||||||||||||||||||||||||||||||||||||||||||||||||||||||
Sbjct  784475  CCGGTCCGTGTCGTTCCGCCGGAAGAGGTCATCGACAGCGAAGCCCTGCTCAAGGGCCGT  784534

Query  121     CGTGAAATCCTGATCCAGCACGGCGACCGCTTCTATCGCCTGCGGCACACCAGCAACGAC  180
               ||||||||||||||||||||||||||||||||||||||||||||||||||||||||||||
Sbjct  784535  CGTGAAATCCTGATCCAGCACGGCGACCGCTTCTATCGCCTGCGGCACACCAGCAACGAC  784594

Query  181     AAGCTGATCCTGACCAAGTA  200
               ||||||||||||||||||||
Sbjct  784595  AAGCTGATCCTGACCAAGTA  784614
```

Stenotrophomonas maltophilia D457 complete genome

GenBank: HE798556.1

```
FEATURES             Location/Qualifiers
     source          1..200
                     /organism="Stenotrophomonas maltophilia D457"
                     /mol_type="genomic DNA"
                     /strain="D457"
                     /db_xref="taxon:1163399"
                     /country="Spain:Mostoles"
     gene            7..>200
                     /locus_tag="SMD_0678"
     CDS             7..>200
                     /locus_tag="SMD_0678"
                     /codon_start=1
                     /transl_table=11
                     /product="Hemin uptake protein"
```

Electron transfer flavoprotein, beta subunit (ETFb)

Description	Max score	Total score	Query cover	E value	Ident	Accession
Stenotrophomonas maltophilia D457 complete genome	398	398	100%	3e-107	100%	HE798556.1
Stenotrophomonas maltophilia R551-3, complete genome	392	392	100%	1e-105	99%	CP001111.1
Stenotrophomonas maltophilia strain ISMMS2, complete genome	381	381	100%	3e-102	99%	CP011305.1
Stenotrophomonas maltophilia strain ISMMS2R, complete genome	381	381	100%	3e-102	99%	CP011306.1
PREDICTED: Pantholops hodgsonii electron transfer flavoprotein subunit beta, mitochondria	370	370	100%	7e-99	98%	XM_005963657.1
Stenotrophomonas maltophilia JV3, complete genome	370	370	100%	7e-99	98%	CP002986.1
Stenotrophomonas maltophilia K279a complete genome, strain K279a	364	364	100%	3e-97	97%	AM743169.1
Stenotrophomonas maltophilia strain ISMMS3, complete genome	353	353	100%	7e-94	96%	CP011010.1
Stenotrophomonas maltophilia strain 13637 genome	353	353	100%	7e-94	96%	CP008838.1

Stenotrophomonas maltophilia D457 complete genome

Sequence ID: emb|HE798556.1| **Length:** 4769156 **Number of Matches:** 1

Range 1: 621185 to 621399 GenBank Graphics ▼ Next Match ▲ Previous Match

Score	Expect	Identities	Gaps	Strand
398 bits(215)	3e-107	215/215(100%)	0/215(0%)	Plus/Minus

```
Query  1       CCTGGAAACGCTGGAAGTCGATCTGCCGGCCGTGGTCACCACCGACCTGCGCCTGAACGA  60
               ||||||||||||||||||||||||||||||||||||||||||||||||||||||||||||
Sbjct  621399  CCTGGAAACGCTGGAAGTCGATCTGCCGGCCGTGGTCACCACCGACCTGCGCCTGAACGA  621340

Query  61      GCCGCGCTTCATCAAGCTGCCGGACATCATGAAGGCCAAGGCCAAGCCGCTGGAGACCCT  120
               ||||||||||||||||||||||||||||||||||||||||||||||||||||||||||||
Sbjct  621339  GCCGCGCTTCATCAAGCTGCCGGACATCATGAAGGCCAAGGCCAAGCCGCTGGAGACCCT  621280

Query  121     GCAGCTGGCCGACCTCGGCGTTGAAGCCGCCGACACCTTCAAGACCACCCAGTACGCCGC  180
               ||||||||||||||||||||||||||||||||||||||||||||||||||||||||||||
Sbjct  621279  GCAGCTGGCCGACCTCGGCGTTGAAGCCGCCGACACCTTCAAGACCACCCAGTACGCCGC  621220

Query  181     GCCGTCCAAGCGCAGCAAGGGTGTGATGGTCAAGG  215
               |||||||||||||||||||||||||||||||||||
Sbjct  621219  GCCGTCCAAGCGCAGCAAGGGTGTGATGGTCAAGG  621185
```

Stenotrophomonas maltophilia D457 complete genome

GenBank: HE798556.1

```
FEATURES             Location/Qualifiers
     source          1..215
                     /organism="Stenotrophomonas maltophilia D457"
                     /mol_type="genomic DNA"
                     /strain="D457"
                     /db_xref="taxon:1163399"
                     /country="Spain:Mostoles"
     gene            complement(<1..>215)
                     /gene="etfB"
                     /locus_tag="SMD_0543"
     CDS             complement(<1..>215)
                     /gene="etfB"
                     /locus_tag="SMD_0543"
                     /codon_start=1
                     /transl_table=11
                     /product="Electron transfer flavoprotein, beta subunit"
```

Ferric siderophore transport system, periplasmic binding protein (TonB)

Description	Max score	Total score	Query cover	E value	Ident	Accession
Stenotrophomonas maltophilia D457 complete genome	564	564	100%	4e-157	99%	HE798556.1
Stenotrophomonas maltophilia strain 13637 genome	525	525	100%	2e-145	97%	CP008838.1
Stenotrophomonas maltophilia K279a complete genome, strain K279a	525	525	100%	2e-145	97%	AM743169.1
Stenotrophomonas maltophilia JV3, complete genome	514	514	100%	4e-142	96%	CP002986.1
Stenotrophomonas maltophilia strain ISMMS2, complete genome	475	475	100%	2e-130	94%	CP011305.1
Stenotrophomonas maltophilia strain ISMMS2R, complete genome	475	475	100%	2e-130	94%	CP011306.1
Stenotrophomonas maltophilia strain ISMMS3, complete genome	464	464	100%	5e-127	93%	CP011010.1
Stenotrophomonas maltophilia R551-3, complete genome	453	453	99%	1e-123	93%	CP001111.1

Stenotrophomonas maltophilia D457 complete genome

Sequence ID: emb|HE798556.1| **Length:** 4769156 **Number of Matches:** 1

Range 1: 4553337 to 4553650 GenBank Graphics ▼ Next Match ▲ Previous Match

Score	Expect	Identities	Gaps	Strand
564 bits(305)	4e-157	311/314(99%)	0/314(0%)	Plus/Plus

```
Query  1        CGCGAGAACCGCATGTATGCGCCGGCCGGCGACAACGCCATCGAGTACTACCTCGCCCTG  60
                ||||||||||||||||| ||||||||||||||||||||||||||||||||||||||||||
Sbjct  4553337  CGCGAGAACCGCATGTACGCGCCGGCCGGCGACAACGCCATCGAGTACTACCTCGCCCTG  4553396

Query  61       CGCGACAAGACCCCGGACGACGCCTCGGTGAAGAGCGCGCTGACCGACCTGCTGCCGTAC  120
                ||||||||||||||||||||||||||||||||||||||||||||||||||||||||||||
Sbjct  4553397  CGCGACAAGACCCCGGACGACGCCTCGGTGAAGAGCGCGCTGACCGACCTGCTGCCGTAC  4553456

Query  121      ACCCTGATCGCCGCCGAGCAGCACCTGGGGCGCGAGGACTACACCGAAGCGCAGCGACTG  180
                |||||||||||||||||||||||||||||||||||||||||||||||||||||||| |||
Sbjct  4553457  ACCCTGATCGCCGCCGAGCAGCACCTGGGGCGCGAGGACTACACCGAAGCGCAGCGGCTG  4553516

Query  181      GTGGCACTGATCGAGAAGGTGGATGCATCCGCGCCGGCCCTGCCGCGCCTGAAGGACGGC  240
                ||||| ||||||||||||||||||||||||||||||||||||||||||||||||||||||
Sbjct  4553517  GTGGCGCTGATCGAGAAGGTGGATGCATCCGCGCCGGCCCTGCCGCGCCTGAAGGACGGC  4553576

Query  241      CTGGCCAAGGGCGTGCAGAACGCGGCCAAGCGCACCGAGGCCGAAGCCGAGAAGGCCAAG  300
                ||||||||||||||||||||||||||||||||||||||||||||||||||||||||||||
Sbjct  4553577  CTGGCCAAGGGCGTGCAGAACGCGGCCAAGCGCACCGAGGCCGAAGCCGAGAAGGCCAAG  4553636

Query  301      AAGGACGCCGAGGA  314
                ||||||||||||||
Sbjct  4553637  AAGGACGCCGAGGA  4553650
```

Stenotrophomonas maltophilia D457 complete genome

GenBank: HE798556.1

```
FEATURES             Location/Qualifiers
     source          1..314
                     /organism="Stenotrophomonas maltophilia D457"
                     /mol_type="genomic DNA"
                     /strain="D457"
                     /db_xref="taxon:1163399"
                     /country="Spain:Mostoles"
     gene            <1..>314
                     /locus_tag="SMD_4053"
     CDS             <1..>314
                     /locus_tag="SMD_4053"
                     /codon_start=1
                     /transl_table=11
                     /product="Ferric siderophore transport system, periplasmic
                     binding protein TonB"
```

Ferric siderophore transport system, biopolymer transport protein (ExbB)

Description	Max score	Total score	Query cover	E value	Ident	Accession
Stenotrophomonas maltophilia K279a complete genome, strain K279a	459	459	100%	2e-125	99%	AM743169.1
Stenotrophomonas maltophilia strain 13637 genome	453	453	100%	8e-124	99%	CP008838.1
Stenotrophomonas maltophilia strain ISMMS2, complete genome	420	420	100%	8e-114	97%	CP011305.1
Stenotrophomonas maltophilia strain ISMMS2R, complete genome	420	420	100%	8e-114	97%	CP011306.1
Stenotrophomonas maltophilia JV3, complete genome	375	375	100%	2e-100	94%	CP002986.1
Stenotrophomonas maltophilia strain ISMMS3, complete genome	370	370	100%	8e-99	93%	CP011010.1
Stenotrophomonas maltophilia D457 complete genome	364	364	100%	4e-97	93%	HE798556.1
Stenotrophomonas maltophilia R551-3, complete genome	353	353	100%	8e-94	92%	CP001111.1

Stenotrophomonas maltophilia K279a complete genome, strain K279a

Sequence ID: emb|AM743169.1| **Length:** 4851126 **Number of Matches:** 1

Range 1: 1703311 to 1703561 GenBank Graphics ▼ Next Match ▲ Previous Match

Score	Expect	Identities	Gaps	Strand
459 bits(248)	2e-125	250/251(99%)	0/251(0%)	Plus/Plus

```
Query  1        GAGCGTTTCTGGTCCCTTCGGCGGACTGAAGTGCTGCCGCCCGGCCTCGGCCAGGAAGTG  60
                ||||||||||||||||||||||||||||||||||||||||||||||||||||||||||||
Sbjct  1703311  GAGCGTTTCTGGTCCCTTCGGCGGACTGAAGTGCTGCCGCCCGGCCTCGGCCAGGAAGTG  1703370

Query  61       CGCAACTGGGCCGCCCGCGGCAAGCTCGACCCGGCCCACCTGCAGACCCTGCGGGCGAAC  120
                |||||||||||||| |||||||||||||||||||||||||||||||||||||||||||||
Sbjct  1703371  CGCAACTGGGCCGCTCGCGGCAAGCTCGACCCGGCCCACCTGCAGACCCTGCGGGCGAAC  1703430

Query  121      TCGCCGCTGGGCGCGCTGCTGGCTGCCGCACTGGAAGCCCGCAACCGCCCGCGCGACCAG  180
                ||||||||||||||||||||||||||||||||||||||||||||||||||||||||||||
Sbjct  1703431  TCGCCGCTGGGCGCGCTGCTGGCTGCCGCACTGGAAGCCCGCAACCGCCCGCGCGACCAG  1703490

Query  181      ATCCGCGAGCGCATCGAAGACACCGGCCGCCACCTGGTGCACCGCATGGAGCGATTCCTG  240
                ||||||||||||||||||||||||||||||||||||||||||||||||||||||||||||
Sbjct  1703491  ATCCGCGAGCGCATCGAAGACACCGGCCGCCACCTGGTGCACCGCATGGAGCGATTCCTG  1703550

Query  241      AACGCACTGGG  251
                |||||||||||
Sbjct  1703551  AACGCACTGGG  1703561
```

Stenotrophomonas maltophilia K279a complete genome, strain K279a

GenBank: AM743169.1

```
FEATURES             Location/Qualifiers
     source          1..251
                     /organism="Stenotrophomonas maltophilia K279a"
                     /mol_type="genomic DNA"
                     /strain="K279a"
                     /db_xref="taxon:522373"
                     /country="United Kingdom"
     gene            <1..>251
                     /locus_tag="Smlt1638"
     CDS             <1..>251
                     /locus_tag="Smlt1638"
                     /codon_start=1
                     /transl_table=11
                     /product="putative biopolymer transport protein"
```

Hemin transport protein (Htp)

Description	Max score	Total score	Query cover	E value	Ident	Accession
Stenotrophomonas maltophilia K279a complete genome, strain K279a	427	427	100%	4e-116	99%	AM743169.1
Stenotrophomonas maltophilia strain 13637 genome	394	394	100%	4e-106	97%	CP008838.1
Stenotrophomonas maltophilia R551-3, complete genome	366	366	98%	9e-98	95%	CP001111.1
Stenotrophomonas maltophilia strain ISMMS2, complete genome	361	361	100%	4e-96	94%	CP011305.1
Stenotrophomonas maltophilia strain ISMMS2R, complete genome	361	361	100%	4e-96	94%	CP011306.1
Stenotrophomonas maltophilia strain ISMMS3, complete genome	350	350	100%	1e-92	94%	CP011010.1
Stenotrophomonas maltophilia D457 complete genome	342	342	99%	2e-90	93%	HE798556.1
Stenotrophomonas maltophilia JV3, complete genome	322	322	100%	2e-84	91%	CP002986.1

Stenotrophomonas maltophilia strain ISMMS2, complete genome

Sequence ID: gb|CP011305.1| **Length:** 4509724 **Number of Matches:** 1

Range 1: 735723 to 735957 GenBank Graphics ▼ Next Match ▲ Previous Match

Score	Expect	Identities	Gaps	Strand
361 bits(195)	4e-96	222/235(94%)	1/235(0%)	Plus/Plus

```
Query  1       CACCGTGTTGTGCCTGTACCACGCCGACGGCAACGAGCTGGGCGGCTGGCGCCAGGCCGT  60
               |||||||||||||||||||||||||||||||||||||||||| |||||||||||||||||
Sbjct  735723  CACCGTGTTGTGCCTGTACCACGCCGACGGCAACGAGCTGGGTGGCTGGCGCCAGGCCGT  735782

Query  61      ACGCGTACACGCCTGCCAGGGCGTGGACAGCGAGGGCCTGCGCGAAAGCCTGTGCTTCCT  120
               |||||| ||||||||||||||||||||||| || ||||||||||| |||||||| |||||
Sbjct  735783  ACGCGTGCACGCCTGCCAGGGCGTGGACAGTGAAGGCCTGCGCGAGAGCCTGTGTTTCCT  735842

Query  121     TGATGGCCGCGGCCGCTGCGTGTGGCGCCTGTACCTGCTGCCCGACAGCGATTTCCTGGC  180
                ||||||||||||||||||||||||||  |||||||||||||||||||||| ||||||||
Sbjct  735843  CGATGGCCGCGGCCGCTGCGTGTGGCGTTTGTACCTGCTGCCCGACAGCGACTTCCTGGC  735902

Query  181     CTGGGACCGTCTGGTCGCGGCGCTGCCGCCGGGACCGGAACACGATAGCGA-GGC  234
               ||||||||| ||||||||||||||||||||||||||||||||||| ||||| |||
Sbjct  735903  CTGGGACCGCCTGGTCGCGGCGCTGCCGCCGGGACCGGAACACGACAGCGATGGC  735957
```

Stenotrophomonas maltophilia strain ISMMS2, complete genome

GenBank: CP011305.1

```
FEATURES             Location/Qualifiers
     source          1..235
                     /organism="Stenotrophomonas maltophilia"
                     /mol_type="genomic DNA"
                     /strain="ISMMS2"
                     /isolation_source="blood"
                     /host="Homo sapiens"
                     /db_xref="taxon:40324"
                     /country="USA: New York, NY"
                     /lat_lon="40.7898553 N 73.9531696 W"
                     /collection_date="2013"
                     /collected_by="The Mount Sinai Hospital"
     gene            <1..>235
                     /locus_tag="YH67_03255"
     CDS             <1..>235
                     /locus_tag="YH67_03255"
                     /inference="EXISTENCE: similar to AA
                     sequence:RefSeq:WP_019661101.1"
                     /note="Derived by automated computational analysis using
                     gene prediction method: Protein Homology."
                     /codon_start=1
                     /transl_table=11
                     /product="Hemin transport protein"
```

TonB-dependent hemin, ferrichrome receptor (FCR)

Description	Max score	Total score	Query cover	E value	Ident	Accession
Stenotrophomonas maltophilia K279a complete genome, strain K279a	691	691	100%	0.0	99%	AM743169.1
Stenotrophomonas maltophilia strain 13637 genome	686	686	100%	0.0	99%	CP008838.1
Stenotrophomonas maltophilia R551-3, complete genome	647	647	100%	0.0	97%	CP001111.1
Stenotrophomonas maltophilia strain ISMMS2, complete genome	636	636	100%	1e-178	97%	CP011305.1
Stenotrophomonas maltophilia strain ISMMS2R, complete genome	636	636	100%	1e-178	97%	CP011306.1
Stenotrophomonas maltophilia JV3, complete genome	233	233	54%	2e-57	87%	CP002986.1
Stenotrophomonas maltophilia D457 complete genome	231	231	58%	6e-57	85%	HE798556.1
Stenotrophomonas maltophilia strain ISMMS3, complete genome	161	161	28%	8e-36	94%	CP011010.1

Stenotrophomonas maltophilia strain 13637 genome

Sequence ID: gb|CP008838.1| **Length:** 4989312 **Number of Matches:** 1

Range 1: 746379 to 746758 GenBank Graphics ▼ Next Match ▲ Previous Match

Score	Expect	Identities	Gaps	Strand
686 bits(371)	0.0	377/380(99%)	0/380(0%)	Plus/Plus

```
Query  1       CGGAAATGAAGGCCGGTATCGAGTTTGGCGAACTCAGCCCGGCGCTGAAGGGCTGGGCGC  60
               |||||||||||||||| |||||||| ||||||||||||||||||||||||||||||||||
Sbjct  746379  CGGAAATGAAGGCCGGCATCGAGTTCGGCGAACTCAGCCCGGCGCTGAAGGGCTGGGCGC  746438

Query  61      TGCGCAGTGCGTTGGCATGGTCGCGTGGTGACAACCGGACCGATGACGAACCGCTGGCCT  120
               ||||||||||||||||||||||||||||||||||||||||||||||||||||||||||||
Sbjct  746439  TGCGCAGTGCGTTGGCATGGTCGCGTGGTGACAACCGGACCGATGACGAACCGCTGGCCT  746498

Query  121     CGGTCGATCCGCTGCGTGGCACGCTGGGCCTGATGTACGACACCGATACCTGGGGCGTCG  180
               ||||||||||||||||||||||||||||||||||||||||||||||||||||||||||||
Sbjct  746499  CGGTCGATCCGCTGCGTGGCACGCTGGGCCTGATGTACGACACCGATACCTGGGGCGTCG  746558

Query  181     AACTGGCCGGTACCTTCGTGCAGCGCAAGAAGCGCCTGCCGCCGCCTGCGGCGCAGACCA  240
               ||||||||||||||||||||||||||||||||||||||||||||||||||||||||||||
Sbjct  746559  AACTGGCCGGTACCTTCGTGCAGCGCAAGAAGCGCCTGCCGCCGCCTGCGGCGCAGACCA  746618

Query  241     ATCCGAACGCACCGGCGCCGTTCGTCTACCAGCCGGCCGGCTACGGCGTGCTGGACCTGA  300
               ||||||||||||||||||||||||||||||||||||||||||||||||||||||||||||
Sbjct  746619  ATCCGAACGCACCGGCGCCGTTCGTCTACCAGCCGGCCGGCTACGGCGTGCTGGACCTGA  746678

Query  301     TGGCGCACTGGAACTTCGCGCCGGGTGCCACCTTCAATGTCGGCGTGTTCAACCTGGCCG  360
               ||||||||||||||||||||||||||||||||||||||||||||||||||||||||||||
Sbjct  746679  TGGCGCACTGGAACTTCGCGCCGGGTGCCACCTTCAATGTCGGCGTGTTCAACCTGGCCG  746738

Query  361     ACAAGCGCTACATCGAATGG  380
               ||||||| ||||||||||||
Sbjct  746739  ACAAGCGTTACATCGAATGG  746758
```

Stenotrophomonas maltophilia strain 13637 genome

GenBank: CP008838.1

```
FEATURES             Location/Qualifiers
     source          1..380
                     /organism="Stenotrophomonas maltophilia"
                     /mol_type="genomic DNA"
                     /strain="13637"
                     /db_xref="taxon:40324"
     gene            <1..>380
                     /locus_tag="DP16_685"
     CDS             <1..>380
                     /locus_tag="DP16_685"
                     /note="tonB-hemlactrns: TonB-dependent
                     hemoglobin/transferrin/lactoferrin receptor family
                     protein"
                     /codon_start=1
                     /transl_table=11
                     /product="TonB-dependent
                     hemoglobin/transferrin/lactoferrin receptor family protein"
```

Predicted dye-decolorizing peroxidase, encapsulated subgroup (DyP)

Description	Max score	Total score	Query cover	E value	Ident	Accession
Stenotrophomonas maltophilia D457 complete genome	405	405	100%	2e-109	95%	HE798556.1
Stenotrophomonas maltophilia K279a complete genome, strain K279a	405	405	100%	2e-109	95%	AM743169.1
Stenotrophomonas maltophilia strain 13637 genome	394	394	100%	5e-106	94%	CP008838.1
Stenotrophomonas maltophilia JV3, complete genome	387	387	98%	8e-104	94%	CP002986.1
Stenotrophomonas maltophilia strain ISMMS2, complete genome	383	383	100%	1e-102	93%	CP011305.1
Stenotrophomonas maltophilia strain ISMMS2R, complete genome	383	383	100%	1e-102	93%	CP011306.1
Stenotrophomonas maltophilia R551-3, complete genome	383	383	100%	1e-102	93%	CP001111.1
Stenotrophomonas maltophilia strain ISMMS3, complete genome	355	355	100%	2e-94	91%	CP011010.1

Stenotrophomonas maltophilia K279a complete genome, strain K279a

Sequence ID: emb|AM743169.1| **Length:** 4851126 **Number of Matches:** 1

Range 1: 195850 to 196107 GenBank Graphics ▼ Next Match ▲ Previous Match

Score	Expect	Identities	Gaps	Strand
405 bits(219)	2e-109	245/258(95%)	0/258(0%)	Plus/Plus

```
Query  1       GTGCTGAAGGTGAAGGATGACGAGGCGTCGATCGCCAGGGCACGCGAGGTGCTGGCGAGC  60
               ||||||||||||||||||||||||||||||||||||| |||||||||||||||||| |||
Sbjct  195850  GTGCTGAAGGTGAAGGATGACGAGGCGTCGATCGCCAAGGCACGCGAGGTGCTGGCCAGC  195909

Query  61      ACCGATGACCTGATCAAGAACACCGCCATCCGCGAGATCGAGCGTACCTTCACCTGCAAC  120
               ||||| ||||||||||||||||||||||||||||||||||| || |||||||||||||||
Sbjct  195910  ACCGACGACCTGATCAAGAACACCGCCATCCGCGAGATCGAACGCACCTTCACCTGCAAC  195969

Query  121     GTGGCCATCGGCCACCGCGTGTGGCAGCCGCTGGTGGGCTCCACGCCGCCGCGCGAGCTG  180
               ||||||||||||||||||||||||||||||||||||||| ||||||||||||||||| ||
Sbjct  195970  GTGGCCATCGGCCACCGCGTGTGGCAGCCGCTGGTGGGCACCACGCCGCCGCGCGAGTTG  196029

Query  181     AAGCCGTTCCGTGAGATCAAGGGCGCCACCCACACCGCCGTGTCCACGCCCGGTGACCTG  240
                |||||||||| |||||||||||||| |||||||| |||||||||||||| || ||||||
Sbjct  196030  CAGCCGTTCCGCGAGATCAAGGGCGCGACCCACACGGCCGTGTCCACGCCGGGCGACCTG  196089

Query  241     CTGTACCACATCCGTGCA  258
               ||||||||||||||||||
Sbjct  196090  CTGTACCACATCCGTGCA  196107
```

Stenotrophomonas maltophilia K279a complete genome, strain K279a

GenBank: AM743169.1

```
FEATURES             Location/Qualifiers
     source          1..258
                     /organism="Stenotrophomonas maltophilia K279a"
                     /mol_type="genomic DNA"
                     /strain="K279a"
                     /db_xref="taxon:522373"
                     /country="United Kingdom"
     gene            <1..>258
                     /locus_tag="Smlt0187"
     CDS             <1..>258
                     /locus_tag="Smlt0187"
                     /note="similarity:fasta; with=UniProt:Q2UBB0
                     (EMBL:AP007162;); Aspergillus oryzae.; Predicted
                     iron-dependent peroxidase.; length=315; id 62.215%;
                     ungapped id 62.215%; E()=1e-70; 307 aa overlap; query
                     18-324; subject 8-314"
                     /codon_start=1
                     /transl_table=11
                     /product="putative peroxidase"
```

Ferric uptake regulation protein FUR (Fur)

Description	Max score	Total score	Query cover	E value	Ident	Accession
Stenotrophomonas maltophilia JV3, complete genome	449	449	100%	1e-122	96%	CP002986.1
Stenotrophomonas maltophilia K279a complete genome, strain K279a	449	449	100%	1e-122	96%	AM743169.1
Stenotrophomonas maltophilia strain 13637 genome	438	438	100%	2e-119	95%	CP008838.1
Stenotrophomonas maltophilia R551-3, complete genome	427	427	100%	5e-116	94%	CP001111.1
Stenotrophomonas maltophilia strain ISMMS3, complete genome	416	416	100%	1e-112	94%	CP011010.1
Stenotrophomonas maltophilia strain ISMMS2, complete genome	411	411	100%	5e-111	93%	CP011305.1
Stenotrophomonas maltophilia strain ISMMS2R, complete genome	411	411	100%	5e-111	93%	CP011306.1
Stenotrophomonas rhizophila strain DSM14405 genome	405	405	100%	2e-109	93%	CP007597.1
Stenotrophomonas maltophilia D457 complete genome	388	388	100%	2e-104	92%	HE798556.1

Stenotrophomonas maltophilia JV3, complete genome

Sequence ID: gb|CP002986.1| **Length:** 4544477 **Number of Matches:** 1

Range 1: 1789191 to 1789469 GenBank Graphics ▼ Next Match ▲ Previous Match

Score	Expect	Identities	Gaps	Strand
449 bits(243)	1e-122	268/280(96%)	2/280(0%)	Plus/Plus

```
Query  1        TGACCGCCGAAGACATCTACCGCCAGCTGCTGGAGCACGGCGACGAGATCGGCCTGGCCA  60
                ||||||||||||||||||||||||||||||||||||||||||||||||||||||||||||
Sbjct  1789191  TGACCGCCGAAGACATCTACCGCCAGCTGCTGGAGCACGGCGACGAGATCGGCCTGGCCA  1789250

Query  61       CGGTGTACCGGGTGCTGACCCAGTTCGAGGCCGCCGGCCTCGTGCTCAAGCACAATTTCG  120
                ||||||||||||||||||||||||||||||||||||||||||||||||||||||| ||||
Sbjct  1789251  CGGTGTACCGGGTGCTGACCCAGTTCGAGGCCGCCGGCCTCGTGCTCAAGCACAACTTCG  1789310

Query  121      AGGGCGGCCAGGCGGTCTACGAACTGGACCGTGGCGGCCACCACGACCACATGGTCGACG  180
                | ||||||||||| |||||||| |||||||||||||||||||||||||||||||||||||
Sbjct  1789311  AAGGCGGCCAGGCCGTCTACGAGCTGGACCGTGGCGGCCACCACGACCACATGGTCGACG  1789370

Query  181      TGGACAGTGGCAAGATCATCGAGTTCGAAAGCCACGAGATCGAGGAGCTGCAGCGCA-GA  239
                ||||||| ||||||||||||||||||||||||||||||||||||||||||||||||| ||
Sbjct  1789371  TGGACAGCGGCAAGATCATCGAGTTCGAAAGCCACGAGATCGAGGAGCTGCAGCGCAAGA  1789430

Query  240      TTCGCCGCAGACCATGGCTACGAACTGGAAGAGCACTCGC  279
                 |||| || ||||| |||||||| ||||||||||| ||||
Sbjct  1789431  -TCGCGGCCGACCACGGCTACGAGCTGGAAGAGCATTCGC  1789469
```

Stenotrophomonas maltophilia JV3, complete genome

GenBank: CP002986.1

```
FEATURES             Location/Qualifiers
     source          1..279
                     /organism="Stenotrophomonas maltophilia JV3"
                     /mol_type="genomic DNA"
                     /strain="JV3"
                     /db_xref="taxon:868597"
                     /country="Brazil"
                     /note="isolated from plants"
     gene            <1..>279
                     /locus_tag="BurJV3_1637"
     CDS             <1..>279
                     /locus_tag="BurJV3_1637"
                     /inference="protein motif:PFAM:PF01475"
                     /note="PFAM: Ferric-uptake regulator;
                     KEGG: smt:Smal_1594 Fur family ferric uptake regulator"
                     /codon_start=1
                     /transl_table=11
                     /product="ferric uptake regulator, Fur family"
```
