# Supplementary material for: Putative Iron Acquisition Systems in Stenotrophomonas maltophilia
Source: Molecules. 2018 Aug 16;23(8):2048. doi: 10.3390/molecules23082048 (PMC6222749; doi:10.3390/molecules23082048)
Supplement: Supplementary file 1 [file molecules-23-02048-s001.zip › suppl proof/Table S3 SM IAS.pdf]

Supplementary Table S3: Fold changes in expression under iron-depleted and iron-repleted conditions screened through nCounter Elements for clinical and environmental isolates

Targets (Abbrev.)	Clinical isolates (n=3)				Environmental isolates (n=3)			
	Iron-depleted[a]	Iron-repleted[a]	Fold changes	p-value[b]	Iron-depleted[a]	Iron-repleted[a]	Fold changes	p-value[b]
FeSreg	4.61	1.92	2.40	0.0904	1.67	2.03	-1.22	0.6532
FeSR	23.59	3.84	6.15	**0.0234**	2.94	1.65	1.78	0.5680
FeSS	3.34	4.53	-1.36	0.6867	3.01	2.85	1.05	0.9343
HemO/HO	7.34	2.20	3.34	0.0987	2.25	1.50	1.50	0.4827
HmuV	2.42	1.03	2.34	0.2708	1.08	1.31	-1.22	0.5415
Hyp1	3.93	1.24	3.16	0.1843	2.64	1.67	1.58	0.5320
HmuU	10.61	1.30	8.14	0.0540	2.30	1.95	1.18	0.7832
HmuT	18.60	1.52	12.21	**0.0051**	1.36	1.23	1.10	0.7923
Rp2	4.88	4.30	1.14	0.8149	2.40	2.81	-1.17	0.8189
Hup	65.38	11.98	5.46	**0.0139**	14.64	9.55	1.53	0.6647
ETFb	10.02	4.39	2.28	**0.0104**	6.74	6.16	1.09	0.8241

TonB	2.10	1.03	2.03	**0.0005**	1.71	1.31	1.30	0.6810
ExbB	10.08	2.77	3.64	0.0702	6.82	4.06	1.68	0.2288
Htp	1.33	1.03	1.28	0.3409	1.71	1.04	1.64	0.4518
FCR	9.02	2.42	3.73	0.0856	2.25	1.95	1.15	0.8009
DyP	14.73	6.28	2.35	0.2030	1.32	1.55	-1.17	0.5626
Fur	21.76	6.59	3.30	**0.0027**	3.84	1.87	2.06	0.3325

[a] Average counts of normalized grouped data.
[b] The distribution of the t-statistic is calculated using the Welch-Satterthwaite equation for the degrees of freedom and in the estimation of the 95% confidence limits for observed differential expression between groups.
The p-values in **bold** are consider statistically significant, set at ($p<0.05$)
