# Supplementary material for: Putative Iron Acquisition Systems in Stenotrophomonas maltophilia
Source: Molecules. 2018 Aug 16;23(8):2048. doi: 10.3390/molecules23082048 (PMC6222749; doi:10.3390/molecules23082048)
Supplement: Supplementary file 1 [file molecules-23-02048-s001.zip › suppl proof/Table S4 SM IAS.pdf]

Supplementary Table S4: Mean OD reading for *N. meningitidis*, SM77 and LM10879 under the iron-depleted and iron-repleted conditions

Iron sources	Mean (SD)	F statistics (df) [a]	p-value[b]
N. meningitidis			
Iron-depleted	0.792 (0.582)		
Hemin	1.399 (0.963)		<0.001
Hemoglobin	1.480 (0.873)	24.360 (4)	<0.001
Transferrin	1.653 (0.814)		<0.001
Lactoferrin	1.585 (1.019)		<0.001
**SM77**			
Iron-depleted	2.027 (1.054)		
Hemin	2.224 (1.129)		0.034
Hemoglobin	2.606 (1.154)	21.764 (4)	<0.001
Transferrin	2.756 (1.191)		<0.001
Lactoferrin	2.272 (1.162)		0.012
**LMG10879**			
Iron-depleted	1.346 (0.685)		
Hemin	1.650 (0.840)		0.012
Hemoglobin	2.281 (1.157)	23.549 (4)	<0.001
Transferrin	2.233 (1.142)		<0.001
Lactoferrin	1.733 (1.072)		0.002

[a] One Way RM ANOVA test

[b] Iron-depleted versus respective iron-repleted sources, post-hoc test (Duncan's Method)
