# Supplementary material for: Putative Iron Acquisition Systems in Stenotrophomonas maltophilia
Source: Molecules. 2018 Aug 16;23(8):2048. doi: 10.3390/molecules23082048 (PMC6222749; doi:10.3390/molecules23082048)
Supplement: Supplementary file 1 [file molecules-23-02048-s001.zip › suppl proof/Table S5 SM IAS.pdf]

Supplementary Table S5: nCounter Elements design details for iron acquisition gene expression in *S. maltophilia*.

Targets	Abbrev.	GenBank accession number	Target's position	Probe A T_m (℃)	Probe B T_m (℃)
**Iron siderophore sensor & receptor system**					
Sigma factor ECF subfamily	FeSreg	SMLT_RS18585	196 – 295	90	89
Iron siderophore receptor protein	FeSR	SMLT_RS12940	1484 – 1583	89	89
Iron siderophore sensor protein	FeSS	SMLT_RS18580	120 – 219	92	92
**Heme, hemin uptake and utilization systems in Gram-positives**					
Heme oxygenase, associated with heme uptake	HemO/HO	SMLT_RS18565	318 – 417	90	86
Heme ABC transporter, ATPase component	HmuV	SMLT_RS11325	229 – 328	87	89
Hypothetical protein related to heme utilization	Hyp1	SMLT_RS19415	115 – 214	84	90
Heme ABC transporter, permease protein	HmuU	SMLT_RS11320	556 – 655	88	88
Heme ABC transporter, cell surface heme and hemoprotein receptor	HmuT	SMLT_RS11315	602 – 701	89	89

**Heme, hemin uptake and utilization systems in Gram-negatives**					
Outer membrane receptor proteins, mostly Fe transport	Rp2	SMLT_RS13970	1138 – 1237	87	86
Hemin uptake protein	Hup	SMD_0678	36 – 135	87	89
Electron transfer flavoprotein, beta subunit	ETFb	SMLT_RS03075	316 – 415	89	88
Ferric siderophore transport system, periplasmic binding protein	TonB	SMLT_RS21345	781 – 880	89	89
Ferric siderophore transport system, biopolymer transport protein	ExbB	SMLT_RS07890	483 – 582	84	89
Hemin transport protein	Htp	SMD_0680	147 – 246	89	89
TonB-dependent hemin, ferrichrome receptor	FCR	SMLT_RS03785	848 – 947	89	89
**Encapsulating protein DyP-type peroxidase and ferritin-like protein oligomers**					
Predicted dye-decolorizing peroxidase, encapsulated subgroup	DyP	SMLT_RS00875	346 – 445	86	88
**Oxidative stress related to iron uptake**					
Ferric uptake regulation protein FUR	Fur	SMLT_RS09600	206 – 305	89	87

Abbrev.: Abbreviation; $\mathbf{T_m}$: melting temperature
